# Modelling of radiation damage and beam-induced heating of room-temperature samples at extremely high flux MX beamlines

**DOI:** 10.1107/S2052252525011224

**Published:** 2026-02-12

**Authors:** Martin V. Appleby, Michal W. Kepa, Graeme Winter, Katherine E. McAuley, John H. Beale

**Affiliations:** a Center for Photon Science Paul Scherrer Institut Forschungsstrasse 111 5232 Villigen Switzerland; b Center for Life Sciences Paul Scherrer Institut Forschungsstrasse 111 5232 Villigen Switzerland; c NE-CAT and Department of Chemistry and Chemical Biology, Cornell University, Argonne National Laboratory, Argonne, IL 60439, USA; European Molecular Biology Laboratory, France

**Keywords:** diffraction-limited storage rings, macromolecular crystallography, radiation damage, protein crystal heating, room-temperature macromolecular crystallography, macromolecular machines, X-ray crystallography, serial crystallography

## Abstract

The upgrade of the third-generation synchrotrons to diffraction-limited storage rings will enable a gain of up to two orders of magnitude in brilliance and further enable the creation of multiple macromolecular crystallography (MX) beamlines capable of delivering fluxes in excess of 1 × 10^15^ photons s^−1^, here called extremely high flux (EHF) MX beamlines. These beamlines, such as ID29 at ESRF-EBS, BioCARS at APS-U and MicroMAX at MAX IV, have all been either partly or solely geared towards delivering time-resolved MX experiments at room temperature and realizing microsecond time resolutions. Given the uncharted territory of using dose rates in excess of 50 GGy s^−1^ at many facilities, this article examines some of the expected consequences, suggesting that considerable attention should be paid to beam heating effects for crystals <20 µm exposed to 1 × 10^15^ photons s^−1^. Several strategies have been proposed to mitigate heating effects when high dose rates are required for a time-resolved experiment, including reducing the absorbed dose by increasing the size of the crystal and the beam profile, and explicitly exploiting the motion of the crystal in serial crystallography delivery systems. The model presented here is intended to serve as a useful tool to inform experimental design and support users’ decision making in such cases.

## Abstract 2

Modelling of radiation damage and beam-induced heating at extremely high flux macromolecular crystallography beamlines is presented.

## 1. Introduction

Although many current and upgraded third-generation synchrotrons can and could deliver fluxes in the region of 1 × 10^13^ photons s^−1^ to their macromolecular crystallography (MX) beamlines, such as the European Synchrotron Radiation Facility (ESRF), Advanced Photon Source (APS) and PETRA-III, the lattice upgrade of a medium-sized third-generation ring, such as Diamond Light Source (DLS), Swiss Light Source (SLS) or Soleil, to a diffraction-limited storage ring (DLSR) will improve the flux of the standard monochromatic MX beamline to this range. However, it must be noted that, at the high energies used by MX beamlines (≥12 keV), the photon beam is no longer diffraction-limited (Hettel & Borland, 2013 ▸). A select few of the European beamlines have also taken, or are taking, the opportunity of their facility upgrade to install a double multilayer monochromator (DMM) to broaden the accepted bandwidth (pink beam) and increase the number of photons transmitted to the sample (see Section S1.1 in the supporting information). The principal use case for this extra flux is time-resolved crystallography *via* serial synchrotron crystallography (SSX) (Diederichs & Wang, 2017 ▸).

Assuming the small crystal samples used in serial crystallography (typically ≤25 µm) will need approximately 1 × 10^10^ photons per diffraction pattern, a flux of 1 × 10^12^ to 1 × 10^13^ photons s^−1^ will effectively cap high spatial resolution information at a time resolution of 1–10 ms. A DMM, depending on the allowed bandwidth, can increase the flux by approximately two orders of magnitude, significantly enhancing the potential time resolution. In practice, this means that the necessary 1 × 10^10^ photons, which previously took 1–10 ms to deliver, can now be delivered in 10 µs with a pink beam.

**Figure 1:**
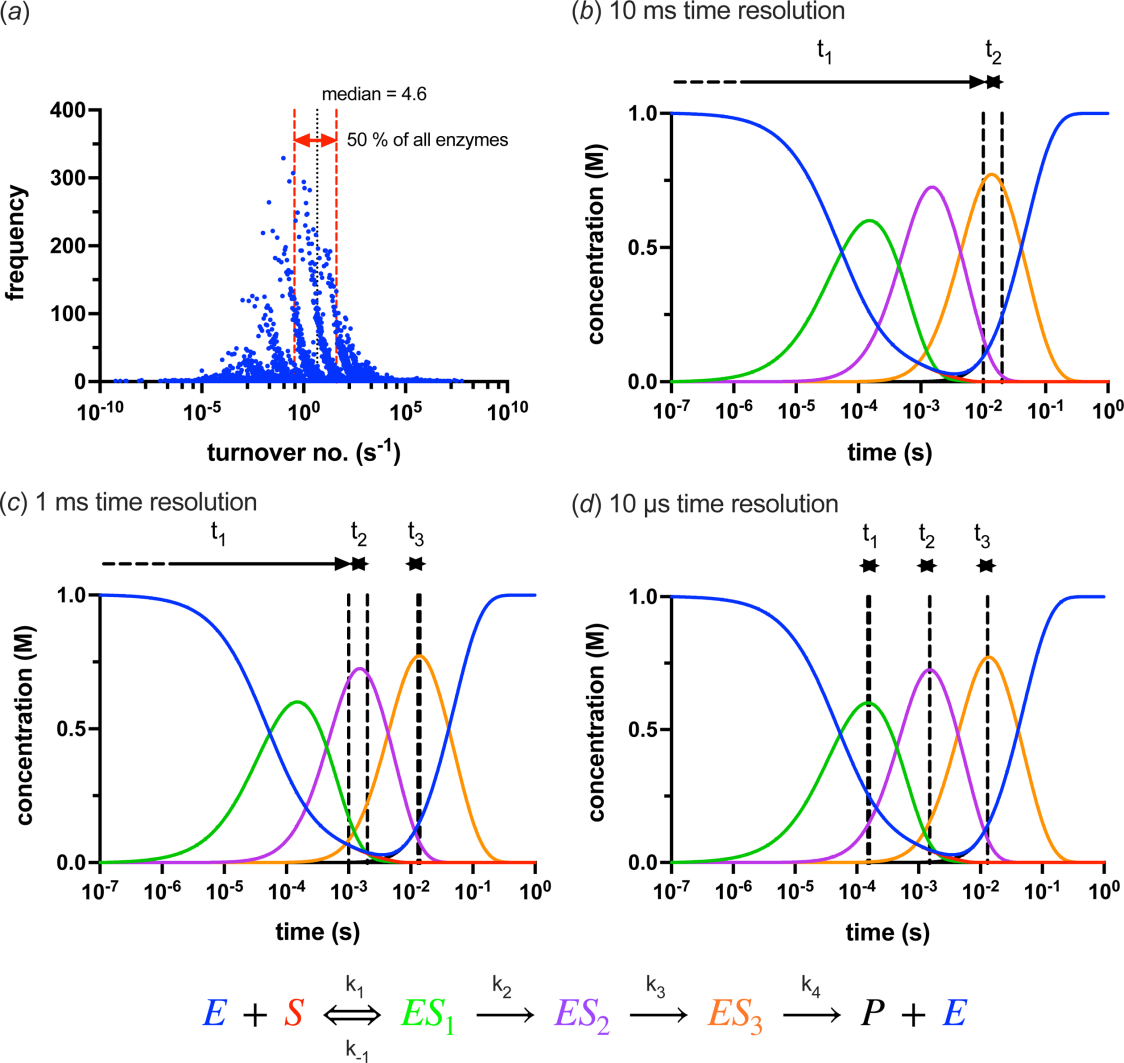
Demonstration of how increased flux can help resolve intermediate reaction species. (*a*) A histogram of turnover numbers compiled in the BRENDA enzyme database (https://www.brenda-enzymes.org). The median turnover number of 4.6 s^−1^ is shown and the range that includes 50% of all enzyme–substrate combinations about this median is also highlighted. (*b*), (*c*) and (*d*) Reaction coordinates for an example enzyme–substrate reaction with three intermediate species and a turnover time of approximately 200 ms (∼5.0 s^−1^ – the reaction scheme is shown at the bottom). The plots show which intermediates (*ES*_1_, *ES*_2_ and *ES*_3_) can be resolved with different detection snapshots: (*b*) 10 ms, (*c*) 1 ms and (*d*) 10 µs, simulating different fluxes of 1 × 10^12^, 1 × 10^13^ and 1 × 10^15^ photons s^−1^, respectively. The rate constant (*k*_1_, *k*_−1_, *k*_2_, *k*_3_, *k*_4_) values used to generate these curves were 10^4.4^, 10^2.4^, 10^3.4^, 10^2.4^ and 10^1.4^, respectively.

The idea of broadening the bandwidth to increase the flux is not new, and particularly not so in time-resolved applications. Beamline ID09 at the ESRF (Wulff *et al.*, 2003 ▸) delivered some of the first Laue time-resolved crystallography results (Šrajer *et al.*, 1996 ▸; Perman *et al.*, 1998 ▸). Later, BioCARS at the APS achieved similar results, accepting bandwidths of 3.0 and 5.5%, and can now reach 7.78 × 10^16^ photons s^−1^ (12 keV, 5.5% bandwidth, 48 bunch mode) after the upgrade to the Advanced Photon Source Upgrade (APS-U) (Graber *et al.*, 2011 ▸; Henning *et al.*, 2024 ▸). New beamlines such as ID29 at the European Synchrotron Radiation Facility – Extremely Bright Source (ESRF-EBS) (Orlans *et al.*, 2025 ▸) and MicroMAX at MAX IV have achieved similar results at lower bandwidths (∼1.0%), albeit with an order of magnitude fewer photons, by installing a DMM. All of these beamlines are examples of what can only be described as extremely high flux (EHF) beamlines, nominally defined here as any unchopped source that can reach approximately 1 × 10^15^ photons s^−1^. The improvement in achievable time resolution that this increased flux enables is highlighted in Fig. 1 ▸.

Since approximately 50% of all enzyme–substrate combinations exhibit turnover rates within the range of 0.35–40 s^−1^ [Fig. 1 ▸(*a*)], an EHF beamline is a powerful tool for capturing structural intermediates across a majority of enzymatic reactions. It is therefore no wonder that more EHF beamlines are in the planning. However, all this extra flux is not without risks.

As has been well described previously (Carugo & Carugo, 2005 ▸; Holton & Frankel, 2010 ▸; Garman, 2010 ▸), radiation damage in protein crystallography usually presents as both site-specific and global damage. The site-specific damage describes localized chemical changes, such as the reduction of metal centres, the breakage of disulfide bonds, and the loss of carboxyl groups from glutamate and aspartate residues. Site-specific damage is either directly due to photon absorption by the protein or ‘indirectly’, *i.e.* ionization by photo-electrons and hydroxyl radicals (Garman, 2010 ▸). A broader classification of indirect site-specific damage is termed ‘secondary’ damage and includes any energy deposition into the protein from the secondary electrons created as a result of the primary absorption event (Garman, 2010 ▸). This definition implicitly includes not only changes in the protein due to chemical ionizations but also the thermalization of energy into the protein itself, *i.e.* crystal heating. Global damage manifests when enough energy is deposited in the crystal to trigger a reduction in the crystalline order and fading of the diffracted intensities. This reduction could be due to a gradual build up of protein-based chemical changes or X-ray–solvent chemistry, such as the production of hydrogen gas (Meents *et al.*, 2010 ▸). For modern synchrotron measurements at approximately 293 K, the main impression an experimenter has of global damage is how rapidly its onset can be observed (Rajendran *et al.*, 2011 ▸; Warkentin *et al.*, 2012 ▸; Leal *et al.*, 2013 ▸; Owen *et al.*, 2012 ▸; Gotthard *et al.*, 2019 ▸; de la Mora *et al.*, 2020 ▸).

Despite this rapid onset, the open question that has never been satisfactorily answered for room-temperature synchrotron experiments is: are the observations of radiation damage onset dependent upon dose rate, *i.e.* can more dose be delivered if that dose is delivered faster? If this statement is true, this is a boon for SSX, as the source can then essentially be treated the same way as an X-ray free-electron laser (XFEL), where more photons will always yield greater diffraction without incurring a radiation damage penalty. Unfortunately, the published literature here is not unequivocal. For dose rates from 0.01 to 10.0 Gy s^−1^, damage appears to be dependent upon dose (Southworth-Davies *et al.*, 2007 ▸; Barker *et al.*, 2009 ▸; Rajendran *et al.*, 2011 ▸), whereas from 0.01 to 1.00 MGy s^−1^ both dependence (Cherezov *et al.*, 2002 ▸; Owen *et al.*, 2012 ▸) and independence (Warkentin *et al.*, 2012 ▸; Leal *et al.*, 2013 ▸) have been observed, and from 1 to 40 MGy s^−1^ only a slight dependence was reported (Cherezov *et al.*, 2002 ▸; de la Mora *et al.*, 2020 ▸). Not breaking this pattern, initial studies that have investigated radiation damage from dose rates achievable at EHF beamlines in the tens of gigagrays per second are also mixed (Orlans *et al.*, 2025 ▸; Gorel *et al.*, 2025 ▸). Nevertheless, despite indications of global damage as a function of dose rate on EHF beamlines, users have collected meaningful data on such beamlines (Stubbs *et al.*, 2024 ▸; Grieco *et al.*, 2024 ▸; Malla *et al.*, 2025 ▸; Sonowal *et al.*, 2025 ▸).

Concerns about the ‘heating effect’ of intense synchrotron radiation X-ray beams on protein crystals are also not new (Blundell & Johnson, 1976 ▸; Helliwell, 1984 ▸), with global damage effects such as unit-cell expansion specifically attributed to them (Ravelli & McSweeney, 2000 ▸; Ravelli *et al.*, 2002 ▸; Cherezov *et al.*, 2002 ▸). The specific concern was that the thermalization of the absorbed photons would heat samples in a 100 K cryogenic stream above the glass transition temperature of ∼155 K (Tilton *et al.*, 1992 ▸; Weik *et al.*, 2001 ▸). Given this concern, the subject garnered significant interest over the years that followed. Kuzay *et al.* (2001 ▸) and Kriminski *et al.* (2003 ▸) used Newton’s law of heat transfer in one and three dimensions, respectively, both modelling the crystal as freely suspended in a gaseous stream. Mhaisekar *et al.* (2005 ▸) used an identical model but employed computational fluid dynamics, whereas Nicholson *et al.* (2001 ▸) used finite element analysis but assumed the crystal was surrounded by solution.

Beam-induced heating has also been experimentally observed, first by the irradiation of glass beads (Snell *et al.*, 2007 ▸) and secondly in the fluorescence of ruby crystals (Warren *et al.*, 2019 ▸). All of these studies agreed that beam-induced heating was not a significant problem for fluxes of approximately 1 × 10^12^ photons s^−1^ and samples cooled to 100 K by a gaseous N_2_ stream. However, as stated by Kriminski *et al.* (2003 ▸), if the flux is increased to that of an EHF beamline (1 × 10^15^ photons s^−1^), this calculation will change and will need to be re-examined.

The aim of the following work is, therefore, to try and model the possible radiation damage limits of these EHF beamlines designed for room-temperature SSX applications, specifically for time-resolved experiments. This analysis was performed in two parts:

(i) modelling the diffraction and dose limits of differently sized protein crystals as a function of the number of incident photons and the size of the crystals, and

(ii) modelling the heating effect as a function of crystal size and flux, while assuming an exposure to a nominal number of photons defined by the previous point.

To achieve the first task, diffraction datasets of human insulin crystals of different sizes and at different fluxes and exposure times were generated using the *nanoBragg* package in *cctbx* (Grosse-Kunstleve *et al.*, 2002 ▸; Holton *et al.*, 2014 ▸). To give some acceptable boundary conditions to facilitate comparisons, it was assumed that, to achieve an ‘ideal’ dataset, a minimum resolution of 2 Å (Holton & Frankel, 2010 ▸) using a CC_1/2_ > 0.3 criterion should be achievable without depositing more than 150 kGy into the crystal [this was taken as the dose limit for insulin (Rajendran *et al.*, 2011 ▸)]. The second task was achieved by creating a new dynamic heating model for beam-induced heating, here called the pulsed adiabatic decay (PAD) model, such that the heating during a given exposure time could be estimated.

The results of these two *in silico* experiments suggest that, firstly, the resolution limits imposed by dose alone (150 kGy in this case) are only problematic for <5 µm crystals. This finding, as one might expect, was true regardless of incident flux. However, this size limit was not true for the beam-induced heating effects as predicted by the PAD model. The extremely high dose rate achievable by beamlines that can achieve micrometre-sized foci of 1.0 × 10^15^ photons s^−1^, such as ID29 and MicroMAX, can become problematic even at beam sizes of 10 µm and 10 µs exposure times. This modelling, though far from perfect, suggests that radiation-induced heating effects may be a serious limitation of EHF beamlines, so care should be taken to mitigate heating effects.

## 2. Methodology

### 2.1. Simulating diffraction images

Datasets of still diffraction images were simulated using the *nanoBragg* package (Holton *et al.*, 2014 ▸) in *cctbx* (Grosse-Kunstleve *et al.*, 2002 ▸). The datasets were modelled using a photon energy of 12.4 keV (λ = 0.9998 Å) with the beam size always equal to the crystal size. Diffraction patterns of human insulin were generated using Protein Data Bank (PDB) code 3i40 (Timofeev *et al.*, 2010 ▸). It should be noted that *nanoBragg* does not explicitly attempt to simulate radiation damage. The *nanoBragg* script used in this study can be found in the Gitea repository at https://gitea.psi.ch/crystal_heating.

A PILATUS 6 Mpixel detector with 2527 × 2463 pixels of 172 × 172 µm (450 µm thick) at a distance of 150 mm was used as the modelled detector. The PILATUS was used due to the current .cbf file format output of *nanoBragg*. The smaller number of larger pixels reduced data volumes and image generation time, making the exercise more computationally feasible. In assessing the limit of the crystal diffraction resolution, the diffraction patterns were simulated to a resolution of 0.5 Å, an approximate 2θ of 45° from the incident beam at 12.4 keV. Background scattering contributions for 35 mm of air were included in the model to represent the beam path from the end of the beam pipe to the beam stop. Water scattering was also included, applying the same thickness as the modelled crystal to approximate the solution surrounding and inside the crystal. It should be noted that this is a non-ideal approximation, particularly for the larger crystals, such as the 100 µm ones, but it was done for consistency between crystal sizes. Given the rarity of 100 µm crystals in serial crystallography applications, these data are included primarily as an edge case point for comparison.

Datasets with beam and crystal sizes of 1, 5, 10, 25, 50 and 100 µm, exposure times of 10, 1 and 0.01 ms, and fluxes of 1 × 10^11^, 1 × 10^12^, 1 × 10^13^ and 1 × 10^15^ photons s^−1^ were each modelled as single-shot still image datasets of 1000 images. Each image was generated at a random orientation. The number of mosaic domains was set to 50 with a spread of 0.1° for all crystal sizes. As the crystal size was changed, the number of unit cells per domain was scaled accordingly. These numbers were calibrated using real diffraction data from 30 µm bovine insulin crystals (see Section S1.2). The energy bandwidth was set to 0.01% [Δ*E*/*E* = 10^−4^ for Si(111)], except for the 10^15^ photons s^−1^ datasets, where it was set to 0.4% to simulate the use of a pink beam from a DMM. All simulated data can be downloaded from the Paul Scherrer Institut’s SciCat data repository at https://doi.psi.ch/detail/10.16907/e9376ecb-dc28-4cc3-9529-8a0933291e8b.

### 2.2. Analysis of simulated data

All datasets were reduced using the *xia2.ssx* package from the *DIALS* suite (Version 3.12.1) (Beilsten-Edmands *et al.*, 2024 ▸) and following standard *DIALS* pipelines with default parameters. Example processing scripts are provided in Section S1.3. Following scaling, the resolution of a given dataset was determined by fitting a tanh function [equation (1)] using the square of the reciprocal of the resolution bin against the CC_1/2_ (Beilsten-Edmands *et al.*, 2020 ▸) [see Fig. S2(*d*) in the supporting information],
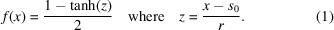


### 2.3. Dose simulations

All dose simulations were performed using *RADDOSE-3D* (Zeldin *et al.*, 2013*a* ▸; Zeldin *et al.*, 2013*b* ▸). The input files used in the investigation are shown in Section S1.4 and the parameters that were changed for each of the different simulations have been labelled. During each of the simulations, 10000000 simphotons (simulated photons) were modelled per crystal and each parameter set was modelled ten times. The average dose exposed region (ADER) was averaged over the ten simulations, and this average was taken as the final dose for a given parameter set (Dickerson *et al.*, 2020 ▸). To take into account photoelectron escape that might be observed in crystals, each crystal was assumed to be in a solution sphere of at least 5 µm, and the potential exchange of photoelectrons with the surrounding solution was enabled (Dickerson & Garman, 2021 ▸). The increased bandwidth of the DMM for the 1 × 10^14^ and 1 × 10^15^ photon fluxes was modelled using the ENERGYFWHM parameter.

### 2.4. Modelling beam heating

#### 2.4.1. Crystal cooling model

The model assumes a transient cooling process and applies Newton’s law of cooling,
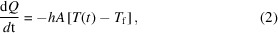
where *h* is the heat transfer coefficient, *A* is the surface area of a cooling body, *Q* is the heat exchanged by the body and the environment, *T*(*t*) is the instantaneous temperature of a body at time *t* and *T*_f_ is the final temperature of the body, *i.e.* the temperature of the environment. Solving the above equation with the boundary condition *T*(*t* = 0) = *T*_i_, which is the initial temperature of the body, yields 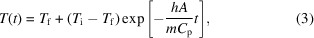
where *m* is the mass of the crystal and *C*_p_ is the heat capacity. The evaluation of the constant *h* is not straightforward, as it depends on the geometry of the body as well as the motion of the gas around it. The model proposed here examines only orders of magnitude of the cooling time and assumes that the heat transfer is identical at each point. Following Fourier’s law in its one-dimensional form (Lienhard & Lienhard, 2020 ▸), we can approximate *h* by assuming laminar boundary layer flow over the surface of a spherical sample (Kriminski *et al.*, 2003 ▸; Lienhard & Lienhard, 2020 ▸) such that
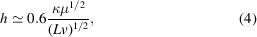
where *v* is the kinematic viscosity of the cryogenic stream (in units of m^2^ s^−1^), κ is the thermal conductivity of the gas (W m^−1^ K^−1^) and μ is the velocity of the gas stream (m s^−1^). *L* is the characteristic sample size along the flow direction and is of the order of *L* ≃ *L*_Xtal_. According to these assumptions, *h* is expected to be independent of the temperature difference between the sample and the environment and only scales with the sample size *L*. Equation (4) holds for sufficiently small samples (less than 500 µm) whereby heat transport is determined by the convective thermal resistance of the boundary layer (Kriminski *et al.*, 2003 ▸).

Although the model is valid according to the stated assumptions, in reality the value of *h* is still an approximation. Therefore, in order to increase the numerical accuracy of the calculated results, the experimental data for ruby crystals from Warren *et al.* (2019 ▸) were used to obtain an empirical scaling factor *s*_exp_ for the value of *h* = 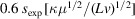 for 100 K samples. Fig. S3(*b*) shows the agreement between the experimental data collected by Warren *et al.* (2019 ▸) and the PAD model, with *s*_exp_ being the only free parameter. Given the reasonable agreement between the model and these data, the scaled *h* values for the different-sized protein crystals could then be back calculated using *s*_exp_ = 1.301.

Unfortunately, the same could not be done for protein crystals at 293 K. Comparable data were collected at ∼293 K by Snell *et al.* (2007 ▸) using glass beads of 1 and 2 mm, which clearly exceed the limit of 500 µm described above and by Kriminski *et al.* (2003 ▸). Consequently, such a case would require modelling the temperature changes governed by heat dissipation within the sample rather than the convective resistance of the boundary layer. Fig. S3(*c*) shows the agreement, or lack thereof, between the 1 mm glass bead raw data and the PAD model. The example decay curve in Fig. S3(*a*) shows the effect of crystal size on the heat decay at 293 K from the differently sized crystals given the above approximations.

#### 2.4.2. Crystal heating model

The beam-induced crystal heating was estimated by modelling the thermal excitation of a given crystal from a photon pulse from a single bunch of electrons and then allowing a relaxation period prior to the next pulse. The parameters used for the heat modelling in this work are shown in Table 1 ▸.

The heating and cooling cycles were repeated for a duration of 1 s to model the point of thermal equilibrium. The key assumption of the model is an ultimate reduction of an idea put forward by Kriminski *et al.* (2003 ▸) that, for very short exposure times, heating can be modelled adiabatically, *i.e.* all of the energy from the dose is assumed to remain in the crystal for the pulse duration. The resulting temperature increase after the pulse can then be given by
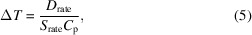
where Δ*T* is the temperature change, *D*_rate_ is the absorbed dose rate (in units of Gy s^−1^), *S*_rate_ is the source repetition rate (Hz) and *C*_p_ is the heat capacity of the crystal (J kg^−1^ K^−1^). Like Kriminski *et al.* (2003 ▸), the model assumes that the crystal is a perfect sphere surrounded by a gaseous stream of 100 K N_2_ or 293 K air [see Fig. 3(*a*)]. Table S3 in the supporting information shows the *C*_p_ calculated by Miyazaki *et al.* (2000 ▸) for lysozyme crystals at 100 and 300 K. In this model, an H_2_O content of 45.7% was assumed, giving *C*_p_ values for 100 and 300 K of 692.5 and 2693.7 J kg^−1^ K^−1^, respectively. The *D*_rate_ was calculated from the ADER with a 1 s exposure time in *RADDOSE-3D*. The thermal decay was modelled as described in Section 2.4.1. Two temperatures were modelled, 100 and 293 K. Given the paucity of data at 293 K, physical constants for 300 K were used instead, where necessary.

Fig. S4 in the supporting information shows the equilibration curves predicted by the PAD model for crystals with diameters of 1, 5, 10, 25, 50 and 100 µm, with fluxes of 1 × 10^12^, 1 × 10^13^, 1 × 10^14^ and 1 × 10^15^ photons s^−1^ at 293 K for a 500 MHz synchrotron with a pulse duration of 50 ps (DLS/SLS in Table 1 ▸). All physical parameters used to generate these curves are given in Table S6 in the supporting information.

## 3. Results

### 3.1. Achievable resolution limits for still image datasets at 293 K

**Figure 2:**
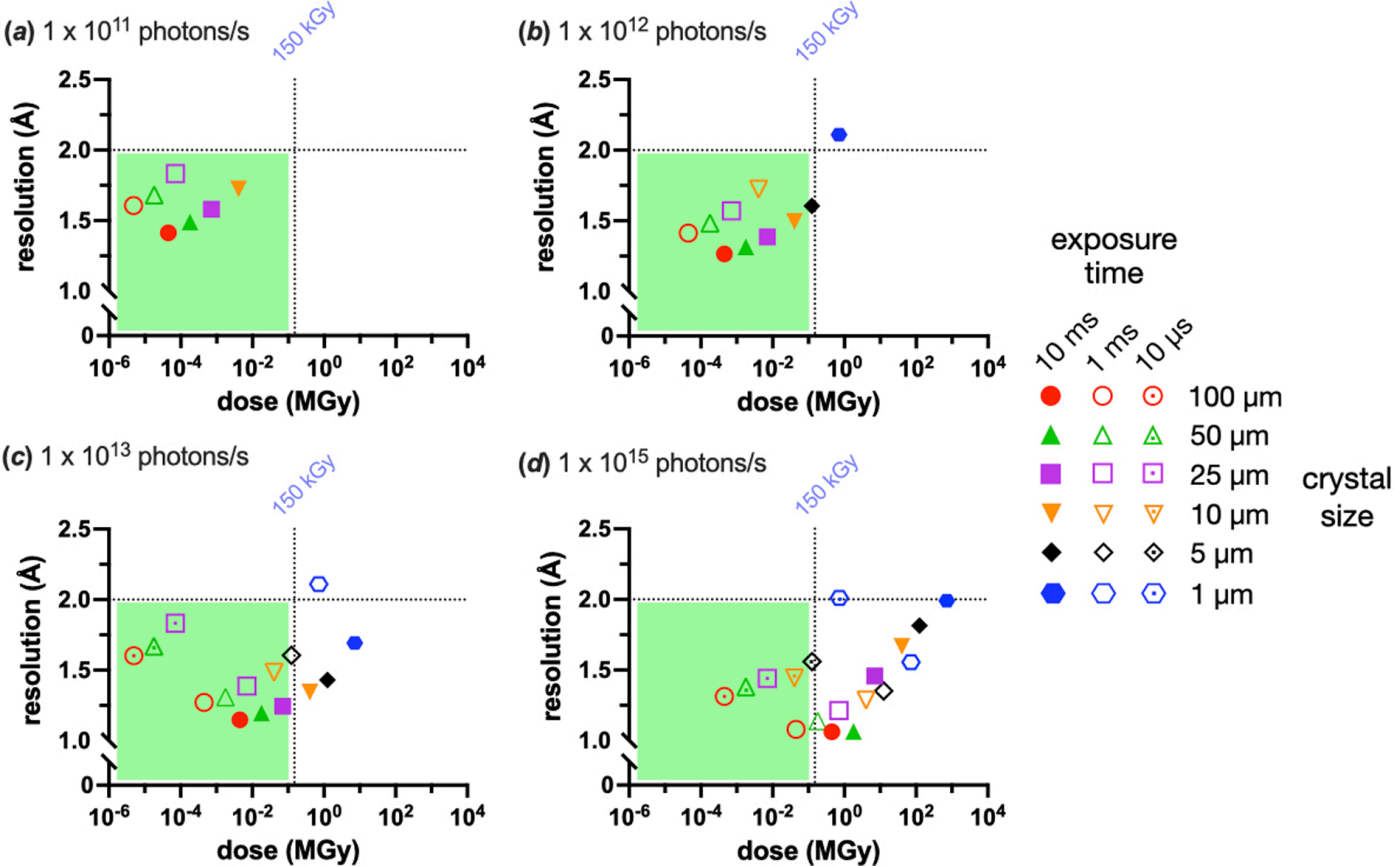
How X-ray dose and crystal size affect achievable resolution for still-image SSX datasets of modelled spherical insulin crystals in a tophat beam of the same size. Six crystal and beam sizes were modelled: 100, 50, 25, 10, 5 and 1 µm, using four different X-ray photon fluxes: (*a*) 1 × 10^11^, (*b*) 1 × 10^12^, (*c*) 1 × 10^13^ and (*d*) 1 × 10^15^ photons s^−1^. The 1 × 10^15^ photons s^−1^ were modelled with an energy bandwidth of 0.4%, mimicking a DMM pink beam. All other fluxes were assumed to be monochromatic at 0.01%. The *x* axes of these plots are given in ADER, as calculated by *RADDOSE-3D* (Zeldin *et al.*, 2013*a* ▸; Zeldin *et al.*, 2013*b* ▸) with the resolution at CC_1/2_ = 0.3 on the *y* axes. The quadrant of each plot where the resolution and dose are below 2 Å and 150 kGy, respectively, is highlighted with a green rectangle. Complete data were modelled but not all datasets could be successfully processed and hence do not appear on the plots.

Fig. 2 ▸ shows how increased flux can affect the achievable resolution of the same protein crystal, in this case human insulin, at different sizes. This modelling explicitly does not take dose rate into account and assumes that there are no dose rate dependent effects. Figs. 2 ▸(*a*) and 2(*b*) show the current situation on many beamlines, a flux of 1 × 10^11^ to 1 × 10^12^ photons s^−1^ and an exposure time of 10 to 100 ms.

With 1 × 10^11^ photons s^−1^ only exposure times of 10 and 1 ms generated processable data, but all were in the ‘green’ zone of better than 2 Å resolution and <150 kGy. An order of magnitude more photons to 1 × 10^12^ photons s^−1^ and an exposure time of 10 µs has still not yet produced any processable data. Crystals of sizes 1 and 5 µm with a 10 ms exposure time generated processable data, but the 1 µm crystals failed to do so at better than 2 Å resolution or less than 150 kGy [Fig. 2 ▸(*b*)]. At 1 × 10^13^ photons s^−1^, 10 µs exposure times yield sub-2 Å resolution data from crystals larger than 25 µm. There are also now enough photons for 1 µm crystals for 1 and 10 ms exposures. However, the necessary dose that allows this is above the 150 kGy limit.

Finally, with 1 × 10^15^ photons s^−1^, the approximate flux of an EHF beamline, 10 µs exposures become possible for 5 µm crystals and larger. There was an issue with the processing of the 10 ms exposure time data here, as the high background and spot intensities limited the correct integration of the highest resolution spots, making the dataset resolution appear lower than it otherwise was. The 1 × 10^15^ photons s^−1^ flux pushed all other crystal sizes below the 2 Å resolution limit but also takes almost all other crystal sizes above the 150 kGy dose limit.

There are, of course, limitations in some of the assumptions of this model. However, this misses the point of the exercise, which is to demonstrate that increased flux, in and of itself, does not improve data collection in small crystals (≤5 µm). All that happens when more photons are used is that the graph’s points shift towards higher resolution, but at the cost of higher dose and higher probable levels of radiation damage. Ultimately, 1 µm crystals never find themselves in the ‘green’ (bottom left) quadrant, and 5 µm crystals are near the dose limit at 125 kGy. If a crystal is more radiation sensitive than insulin, the boundary conditions may move such that the 5 µm crystals are also outside the ‘green zone’. After this exercise, a nominal exposure for the different fluxes was defined as 1 × 10^10^ photons as this was the ‘magic’ value that yielded <2 Å resolution data in <150 kGy for the majority of the different crystal sizes.

### 3.2. Dynamic modelling of beam-induced heating

**Figure 3:**
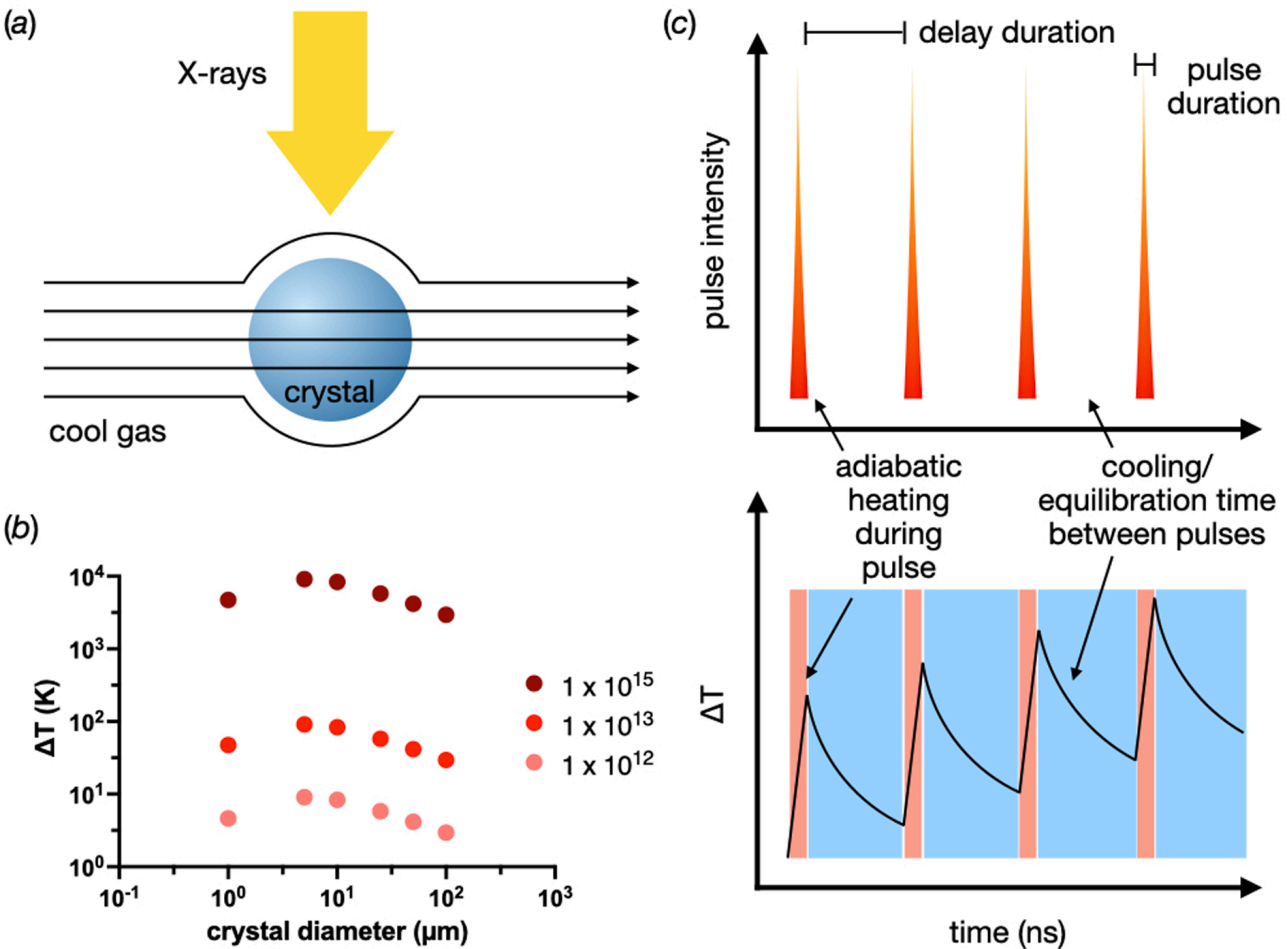
KKT equilibrium and pulsed adiabatic decay (PAD) models. (*a*) Assumption of KKT and PAD models. The crystal is a sphere freely floating in gas flowing around the crystals. (*b*) Thermal equilibria estimated using the KKT model as a function of crystal diameter and flux. (*c*) Idea behind the PAD model. Pure adiabatic heating during the X-ray pulses with cooling, or relaxation time, between pulses.

First, the thermal equilibrium of different crystal sizes in different fluxes was assessed using the model derived by Kriminski *et al.* (2003 ▸) (hereafter referred to as the KKT model) and subsequently adapted by Warren *et al.* (2019 ▸) to include the dose rate instead of the beam intensity. The model assumes that the crystal is a freely floating sphere surrounded by a gaseous stream [Fig. 3 ▸(*a*)]. The point of thermal equilibrium is given by
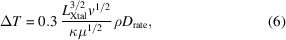
where *L*_Xtal_ is the length of the illuminated volume of the crystal (in units of m), *v* is the kinematic viscosity of the cryogenic stream (m^2^ s^−1^), κ is the thermal conductivity of the gas stream (W m^−1^ K^−1^), μ is the velocity of the gas stream (m s^−1^), ρ is the density of the protein crystal (kg m^−3^) and *D*_rate_ is the dose rate for a given sample (Gy s^−1^). *RADDOSE-3D* ADER values were generated over a 1 s exposure time to calculate *D*_rate_. The additional parameters required are given in Table S4 in the supporting information.

Fig. 3 ▸(*b*) shows the KKT thermal equilibrium estimates for 1 × 10^12^, 1 × 10^13^ and 1 × 10^15^ photons s^−1^ as a function of crystal size; again, the beam size was always matched to the crystal size. As might be expected, a significant increase in the point of thermal equilibrium is observed as a function of flux. However, since there is no time component to equation (6), an estimate of the crystal temperature after a given exposure time cannot be made. Therefore, a new model was formulated to predict the temperature rise as a function of time.

This PAD model is based on an idea from Kriminski *et al.* (2003 ▸) that heat can be adiabatically modelled during short exposure times. This idea is taken to its extreme by the PAD model, as it assumes adiabatic heating for the individual photon pulses of the synchrotron. This heat is then allowed to decay exponentially until the next pulse [Fig. 3 ▸(*c*)]. In line with the KKT model, the crystal was assumed to be a sphere suspended in a gas stream [Fig. 3 ▸(*a*)]. Using these assumptions, protein crystal heating could be modelled as a function of exposure time and dose. The dose rates used to calculate these curves are given in Table S5 in the supporting information, with the highest being 93.6 GGy s^−1^ for the 1 µm crystal with 1 × 10^15^ photons s^−1^. The effects of beam-induced heating were then explored for EHF beamline fluxes during a nominal exposure of 1 × 10^10^ photons.

**Figure 4:**
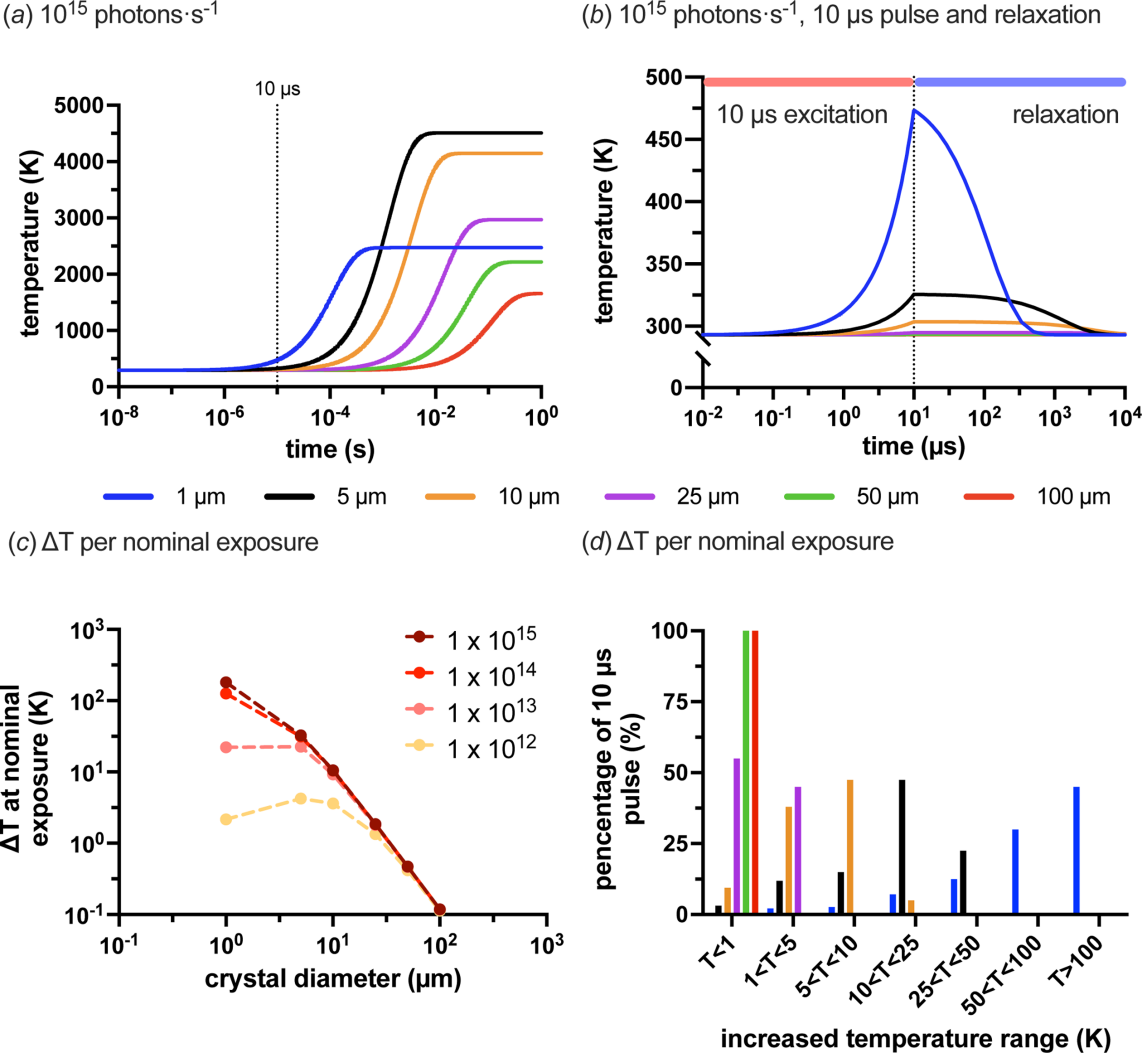
PAD modelled X-ray-beam-induced heating at 293 K at a 500 MHz synchrotron. As per the previous figures, six spherical crystal diameters were modelled: 100, 50, 25, 10, 5 and 1 µm. (*a*) An example of the PAD model for 1 × 10^15^ photons s^−1^ at 293 K. (*b*) A 10 µs exposure of 1 × 10^15^ photons s^−1^ at 293 K and subsequent relaxation back to 293 K. (*c*) Predicted temperature increase per nominal exposure time expected by the PAD model at 293 K (normalized to 1 × 10^10^ photons). The exposure times used were 10 ms, 1 ms, 100 µs and 10 µs for 1 × 10^12^, 1 × 10^13^, 1 × 10^14^ and 1 × 10^15^ photons s^−1^, respectively. (*d*) Predicted proportions of the 10 µs exposure at 1 × 10^15^ photons s^−1^ that a crystal of a given size will occupy different temperature ranges.

Fig. 4 ▸(*a*) shows an example of the thermal equilibrium predicted by the PAD model for different crystal sizes exposed to a flux of 1 × 10^15^ photons s^−1^ at 293 K. The consequence of assuming a photoelectric effect (and enabling the modelling of it in *RADDOSE-3D*) can clearly be observed here. If the photoelectric effect was not enabled when modelling the dose in *RADDOSE-3D*, the equilibrium temperature would continue to rise as the crystal size is reduced. All modelled curves for 1 × 10^12^, 1 × 10^13^, 1 × 10^14^ and 1 × 10^15^photons s^−1^ at 293 K are shown in Fig. S4 in the supporting information [panels (*a*), (*b*), (*c*) and (*d*), respectively]. Fig. 4 ▸(*b*) gives an illustration of the thermodynamics of a single 10 µs exposure and subsequent relaxation time at 293 K and 1 × 10^15^ photons s^−1^ for the different crystal sizes. These plots show the problem presented by 1 × 10^15^ photons s^−1^. Unfortunately, although the flux may have increased by three orders of magnitude, cooling cannot keep up with the increase in energy load.

Fig. 4 ▸(*c*) shows this more clearly by comparing the temperature rise (Δ*T*) in the different crystals for the different fluxes when exposed for nominal times of 10 µs, 100 µs, 1 ms and 10 ms for 1 × 10^15^, 1 × 10^14^, 1 × 10^13^ and 1 × 10^12^ photons s^−1^, respectively (*i.e.* with the same number of photons, 1 × 10^10^). As can be observed, the 100 µm crystals show little change in Δ*T* as a function of the flux for their nominal exposure time. This is also true for the 25 and 50 µm crystals. For crystals smaller than 10 µm, the flux of an EHF beamline becomes more problematic. The PAD model suggests a jump in temperature per exposure of more than 10 K for a flux of ≥1 × 10^13^ photons s^−1^. Assuming that an approximate 10 K increase is within the tolerance of a crystal, all crystals should survive a nominal exposure at 1 × 10^13^ photons s^−1^; however, this cannot be said for 1 × 10^14^ or 1 × 10^15^ photons s^−1^. With a flux of 1 × 10^15^, the 1 and 5 µm crystals increase in temperature by 180 and 32 K, respectively, within the 10 µs exposure. The estimate for the 1 µm crystals is unlikely to be exact, as, beyond the boiling point of water, it is unclear what state the crystal will be in to absorb more heat. Either way, the probable outcome will be the loss of the crystal structure and, therefore, of diffraction.

The other issue this poses is that subsequent diffraction data from a single exposure will ultimately be an ensemble of measurements taken at different temperatures. Fig. 4 ▸(*d*) shows the predicted proportions of a 10 µs exposure time that crystals of different sizes will spend in different temperature ranges. The plot shows that even 25 µm protein crystals may be problematic due to the proportion of the exposure time spent at an elevated temperature. For smaller crystals such as the 1 and 5 µm ones, it is highly likely that the considerable time spent during the exposure at an elevated temperature would result in a resolution-dependent loss of diffraction.

### 3.3. Test cases: modelling beam-induced heating on ID29 and MicroMAX

At the time of writing (December 2025), there are three EHF beamlines that are currently in user operation and capable of performing time-resolved MX experiments: ID29 and ID09 at ESRF-EBS, and MicroMAX at MAX IV. The following examples of how to use the PAD model in practice will focus on ID29 [2.0 × 10^15^ photons s^−1^ (Orlans *et al.*, 2025 ▸)] and MicroMAX (https://www.maxiv.lu.se/beamlines-accelerators/beamlines/micromax) (1.0 × 10^15^ photons s^−1^), as these have been purpose-built for serial crystallography at room temperature. ID09 was not specifically considered, as its last time-resolved MX publication was by Jung *et al.* (2013 ▸), although SSX is still very much alive and well (Tolstikova *et al.*, 2019 ▸). In both the ID29 and MicroMAX cases, the goal is to find data collection parameters that limit beam-induced heating to less than 10 K. More complete instructions on how to use the model and the different options available are given in Section S1.13.

#### 3.3.1. ID29: 5 µm mammalian rhodopsin crystals

In the following example, let us assume that we wish to screen some mammalian rhodopsin microcrystals. The crystals, in this example, only grow to 5 µm cubes and are therefore well suited to the ID29 beam, as it currently has a fixed focus of 4 × 2 µm FWHM (December 2025). The ultimate goal of the experiment is to perform some steady-state and millisecond resolution experiments. The first step is to calculate the deposited dose in 1 s. We suspected that crystal heating might be a problem at higher fluxes, so we decided to calculate the dose for fluxes from 1 × 10^12^ to 1 × 10^15^ photons s^−1^ using *RADDOSE-3D* (see Table S7 in the supporting information).

**Figure 5:**
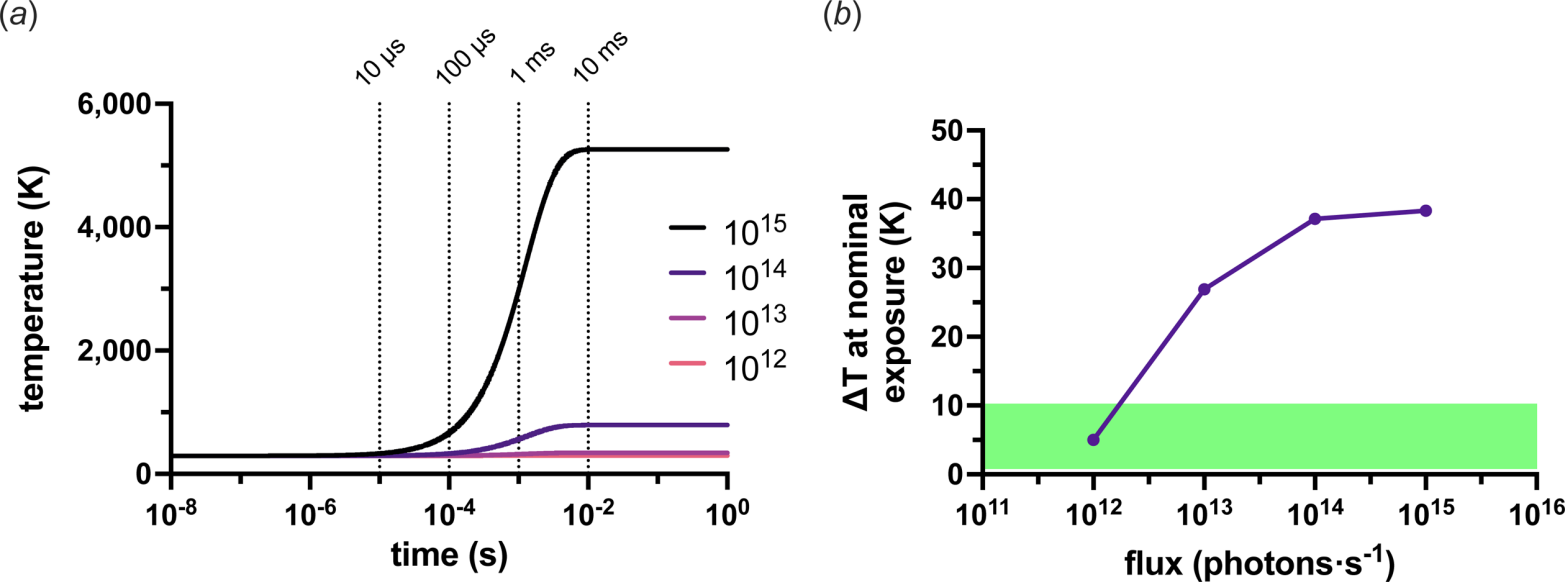
PAD modelling of 5 µm cuboid rhodopsin crystals on ID29. (*a*) Thermal equilibria at different fluxes. (*b*) Predicted temperature rise of the crystals for a nominal exposure of approximately 1 × 10^10^ photons.

Fig. 5 ▸(*a*) shows the PAD modelling from these doses at the different fluxes, and Fig. 5 ▸(*b*) shows the predicted temperature rise given an exposure of approximately 1 × 10^10^ photons. In this case, the PAD model suggests that the safest course of action would be to reduce the incident flux to 1 × 10^12^ photons s^−1^. This dataset could, at the very least, be used as a ‘heating control’ that could be compared with additional datasets at high fluxes to assess the prevalence of heating effects. If these are not found, then higher fluxes and higher time resolutions can be attempted.

#### 3.3.2. MicroMAX: haemoglobin time-resolved experiment

In this case, we wish to conduct a time-resolved experiment on haemoglobin crystals on MicroMAX. We need 10 µs resolution at the highest resolution possible, so we wish to attempt the maximum flux possible, in this case 1 × 10^15^ photons s^−1^. Haemoglobin was chosen since it also contains iron that will further add to the X-ray absorption. However, as luck would have it, the haemoglobin microcrystals in this example grow in cubes that can be easily tuned to any size. Like in the rhodopsin example above, we first calculate the dose for crystal sizes from 5 to 25 µm using *RADDOSE-3D* (Table S8 in the supporting information).

**Figure 6:**
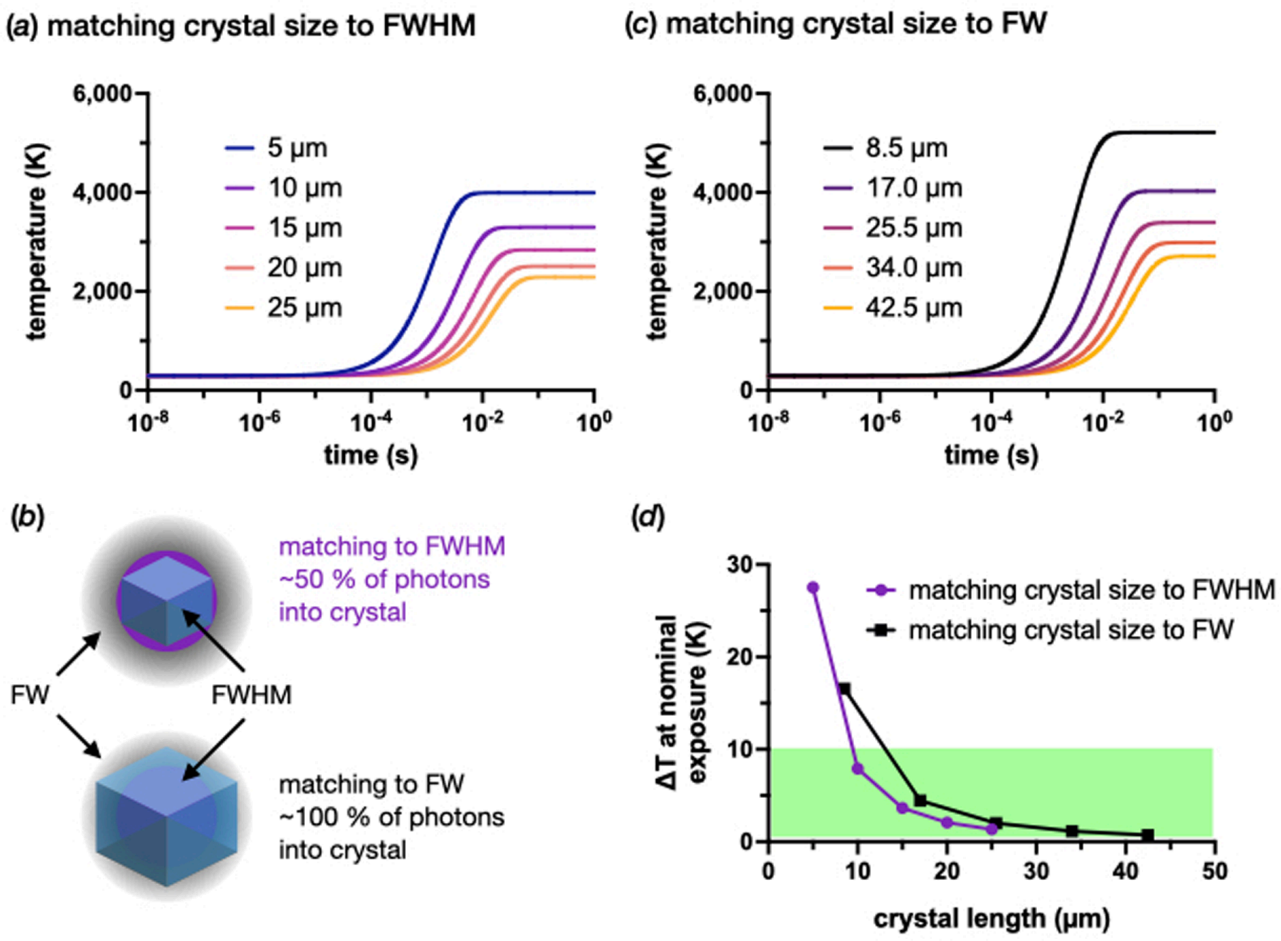
PAD modelling of cuboid haemoglobin crystals on MicroMAX. (*a*) Thermal equilibria at different crystal sizes where the crystal size has been matched to the beam FWHM. (*b*) A to-scale schematic showing the difference between matching the crystal size to the FW or the FWHM. (*c*) Thermal equilibria at different crystal sizes where the crystal size has been matched to the beam FW. (*d*) Comparison of the Δ*T* of the crystal after a 10 µs exposure where either the beam FW or the FWHM matches the crystal size.

In the first run of the PAD model [Fig. 6 ▸(*a*)], the doses were calculated assuming that the FWHM of the Gaussian X-ray beam was matched to the crystal size. This gives slightly strange results when compared with the original modelling in Fig. 4 ▸(*a*) with a tophat profile. The heating appears less pronounced for a Gaussian than for a tophat profile. The reason for this is that the FWHM contains only ∼50% of the photons within a 2D Gaussian profile. This has two consequences in terms of this experiment. Firstly, the effective flux is halved, as fewer photons are actually hitting the crystal, so we should expect reduced diffraction [Fig. 6 ▸(*b*)]. Secondly, the surrounding area of the crystal will now also generate photo-electrons that can travel back into the crystal, essentially negating any gain from the photo-electric effect (Dickerson & Garman, 2021 ▸).

To make better use of the available photons, the crystal sizes were then matched to the 1/*e*^2^ of the Gaussian, denoted the full width (FW; 1.699 × FWHM), and the PAD model was rerun with new dose rates. Fig. 4 ▸(*c*) shows the heating profiles for the FW doses and Fig. 4 ▸(*d*) shows the difference in temperature rise after a 10 µs exposure between the two. Despite increasing the size of the crystals, increasing the number of incident photons hitting the crystals has also increased Δ*T*. However, from 17 µm crystals and larger, the predicted temperature rise over 10 µs is relatively modest. As with the ID29 example above, it would still be an excellent control to collect a dataset at a reduced flux to see whether any differences are apparent between the high and low fluxes.

## 4. Discussion

To summarize the potential of EHF beamlines for time-resolved MX experiments, it is perhaps easiest to say that with great power comes great responsibility. Approximately 1 × 10^15^ photons s^−1^ is an extremely large amount of power, and so an equal or greater amount of responsibility should be exercised in its application. Radiation damage is still a problem that must be navigated on a sample-to-sample basis. This work highlights that, when using an EHF beamline, radiation damage can no longer simply be measured in terms of absorbed dose; the thermalization of that dose now needs to be considered.

Before extrapolating too far from the above modelling, some significant caveats need to be stated. The heating model specifically assumes that the crystal is a stationary sphere and is freely suspended in a dynamic gaseous environment [Fig. 3 ▸(*a*)]. These assumptions were made to simplify the heating and cooling models, but they come at the cost of the transferability of the results. Crystals, particularly those in serial crystallography experiments that EHF beamlines have been built to perform, are almost never freely suspended in air but are immersed in some sort of liquid or viscous medium. This medium, though probably stationary with respect to the crystal and not freely flowing about it like the gas in the model, might be able to conduct heat more efficiently away from the crystal than the solid–gas boundary. However, the model does give a reasonable prediction of the ruby equilibration time and the temperature of that equilibrium for cryo-measurements. It also gives a consistent underestimate of the equilibrium temperature compared with the KKT model. So, if anything, the estimated temperature increases predicted by the PAD model could even be underestimates.

The caveats having been stated, the PAD model and crystal size diffraction modelling suggest that the ability to collect high-resolution data from <5 µm crystals with a 1 × 10^15^ photon flux in 10 µs will be very challenging. Although the total doses of such <5 µm crystals (where the beam size has been matched to the crystal size) may only be in the order of hundreds of kilogray, dose rates of the order of gigagrays per second are necessitated by such exposure times. These are three orders of magnitude higher than recent SSX room-temperature studies into radiation damage in protein crystals (Ebrahim *et al.*, 2019 ▸; Gotthard *et al.*, 2019 ▸; de la Mora *et al.*, 2020 ▸). The experimental evidence for radiation damage at these rates is somewhat mixed. Orlans *et al.* (2025 ▸) found no evidence of lysozyme disulfide bond breakage in an *F*_o_ − *F*_c_ omit map with a dose rate of 9.6 GGy s^−1^, albeit at an unusually low resolution for a lysozyme dataset. On the other hand, Gorel *et al.* (2025 ▸), in an investigation using a heme-protein crystal, at near-identical dose rates and on the same beamline, observed site-specific and global damage occurring within the X-ray exposure. It is possible that the latter observation was partly a function of the use of a heme-containing protein, which are well known for their radiation sensitivity (Berglund *et al.*, 2002 ▸; Beitlich *et al.*, 2007 ▸). However, it is also possible that this discrepancy could be explained by the fact that, at dose rates of gigagrays per second, global damage markers can not only keep pace with site-specific damage indicators but perhaps even outrun them.

It is not clear what impact, if any, a jump in temperature of 10 K would have on a crystal during an exposure. It is very possible that the effects could be minor, as jumps of comparable size have already been initiated by laser pulses with no apparent loss in resolution (Wolff *et al.*, 2023 ▸). Ultimately, any damage is likely to be sample-specific. A good strategy could be, therefore, to collect a lower-dose-rate and lower-flux control dataset as part of any EHF beamline time-resolved experiment by attenuating the beam to 1 × 10^12^ photons s^−1^ and using an exposure time of 10 ms. By comparing the global and site-specific damage indicators with the high-dose-rate high-flux measurement, an estimate of the crystal’s susceptibility to beam heating can be established. Another concern is that the temperature increase could be enough for thermally inducing activation of the very change the experiment is hoping to observe (Baxter *et al.*, 2024 ▸). Again, caution should be practised, and an evaluation of any heating effects should be made on the ground-state structure.

**Figure 7:**
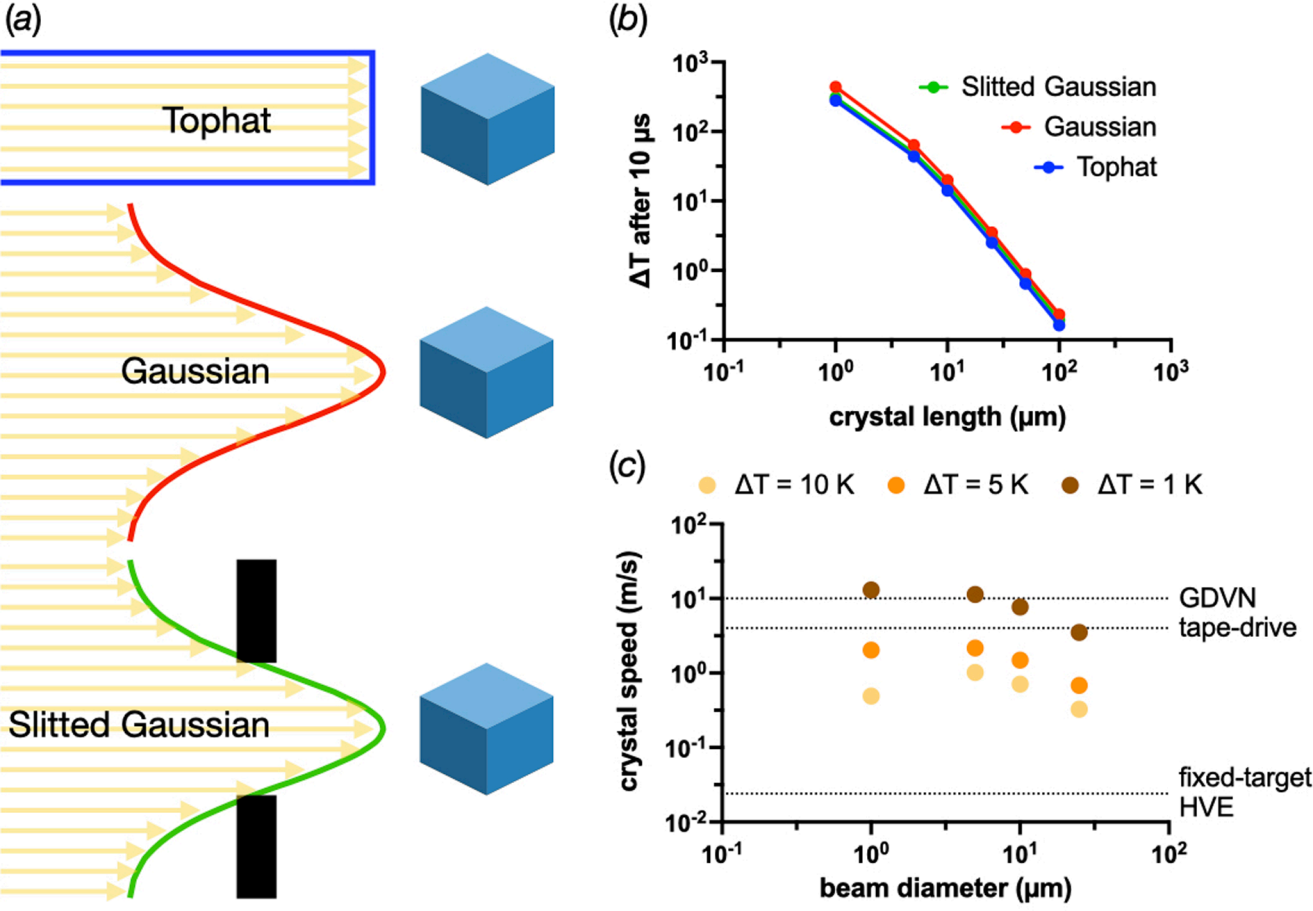
Possible experimental strategies to decrease X-ray-beam-induced heating of small crystals with 1 × 10^15^ photons s^−1^. (*a*) Schematics of the different beam profiles used in the modelling of the data shown in panel (*b*). The crystal size has been matched to the Gaussian FWHM and the tophat collimation. In the slitted Gaussian profile, the slits prevent the Gaussian tails reaching the solution surrounding the crystal. (*b*) The Δ*T* values in differently sized crystals using the different beam profiles shown in panel (*a*). In each case, the number of photons hitting the crystal has been kept the same at 1 × 10^10^. (*c*) Estimates of the necessary crystal speeds through the X-ray beam to ensure maximum Δ*T* values of 10, 5 and 1 K for crystals with a smallest dimension of <25 µm. These estimates assume a crystal translation length per temperature increase limit of the crystal/beam size.

As shown in Fig. 6 ▸, matching the beam width to the crystal can help to reduce heat loads. True tophat beam profiles are challenging to create, particularly with micrometre-sized widths. Unfortunately, the PAD model suggests that only very limited temperature gains can be made by creating a more tophat-like profile by slitting a Gaussian to the FWHM. Deposited doses were calculated in *RADDOSE-3D* for profiles corresponding to the schematics shown in Fig. 7 ▸(*a*) whilst keeping the number of photons hitting the crystal the same at 1 × 10^15^ photons s^−1^. Fig. 7 ▸(*b*) compares the Δ*T* values after 10 µs. The slitted Gaussian results are closer to the tophat profile than to the Gaussian, but the improvements only become meaningful for 5 and 1 µm crystals where the crystals have already been heated by tens and hundreds of kelvin, respectively.

The best strategy, based on this modelling, appears to be to use larger crystals (smallest dimension >20 µm) and match the FW of the beam to these crystals. If crystals with dimensions smaller than 20 µm are necessary, another idea could be to use crystal motion through the beam. All serial crystallography sample delivery systems create a steady stream of new crystals to illuminate. These methods can, therefore, translate the sample through the beam during an exposure and help to spread the dose and limit damage (Khusainov *et al.*, 2024 ▸). Fig. 7 ▸(*c*) shows the translation speeds necessary to limit the heat accumulation in crystals to 1, 5 and 10 K, whose smallest dimension is <25 µm and matched to the beam size. These speeds were estimated by calculating the time taken to reach the given temperature and then dividing the beam size by this time (the time estimates are shown in Table S9 in the supporting information). The beam size and longest crystal dimension need to be considered here, since this approach can only deliver its best results if the crystals are long enough for the required dose to spread along their length, not unlike helical scans in rotation crystallography. It should be noted that this length requirement probably makes limiting the temperature increase to 1 K untenable for crystals whose smallest dimensions are 1, 5 or 10 µm, as this implies the crystal’s longest dimension will need to be 130, 113 and 77 µm, respectively. Crystals of these dimensions are perhaps not unknown, but they are not likely to survive the sample delivery process intact (full length estimates are shown in Table S10 in the supporting information).

Unfortunately, fixed targets and high-viscosity extruders, at least in their current design and implementation, cannot reach the necessary speeds to achieve these gains (Weierstall *et al.*, 2014 ▸; Doak *et al.*, 2023 ▸). Crystal Δ*T* values of approximately <5 K can be achieved using a gas dynamic virtual nozzle (GDVN) (DePonte *et al.*, 2008 ▸; Weierstall *et al.*, 2012 ▸; Weierstall *et al.*, 2014 ▸) with a jet speed of 10 m s^−1^ for all crystal sizes. Tape drive systems (Fuller *et al.*, 2017 ▸; Beyerlein *et al.*, 2017 ▸), though normally run at slower speeds, should also be able to attain suitable velocities to limit the Δ*T* values to <10 K.

## 5. Conclusions

**Figure 8:**
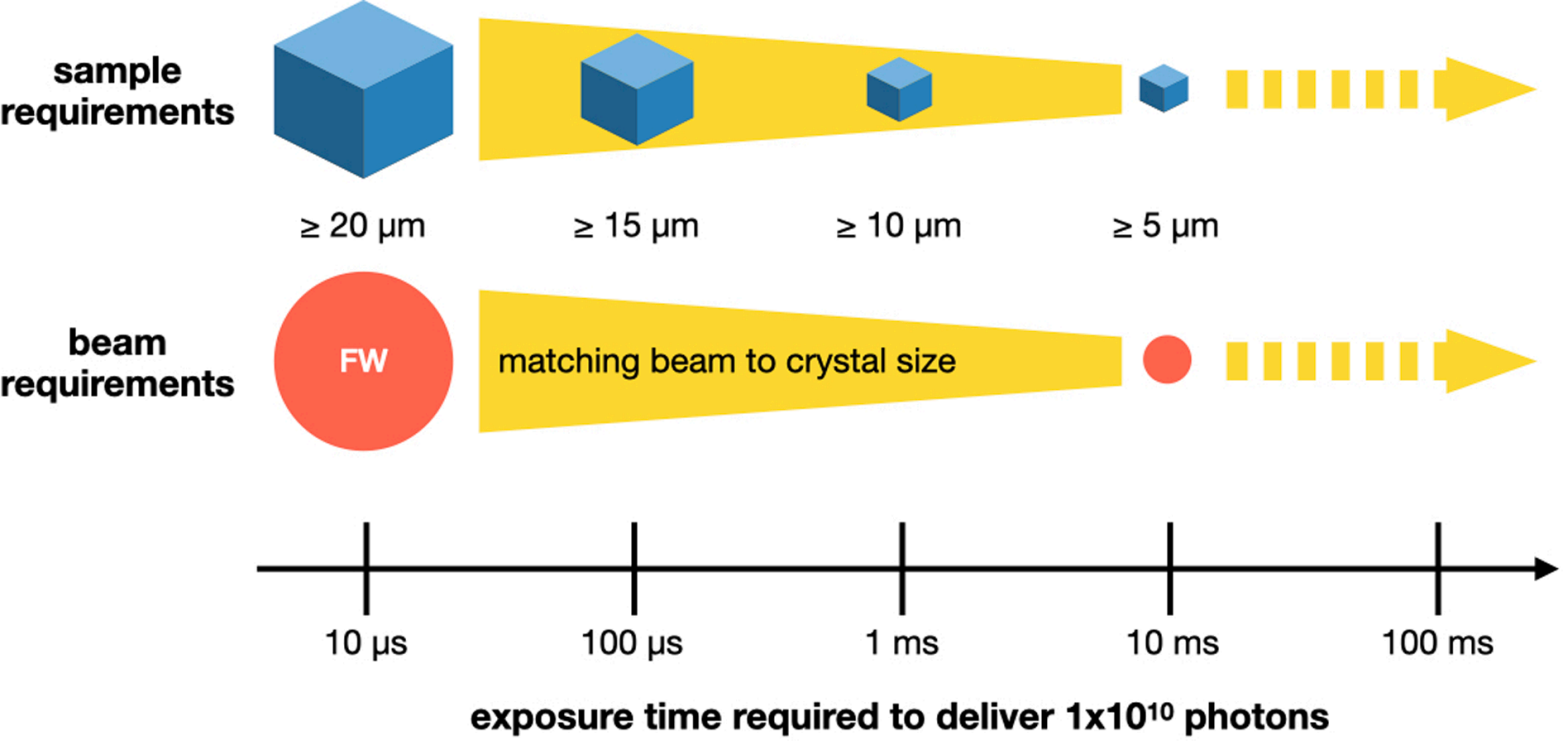
Rough sample and beam requirement guidelines for collecting room-temperature time-resolved data at synchrotron sources based on the PAD model. These numbers assume 1 × 10^10^ photons are required to yield data of an appropriate resolution and should be thought of as lower bounds.

It is clear that there is a serious opportunity presented by an EHF beamline. Even without a DMM, a beamline that has a flux of 1 × 10^13^, suitable additional devices, sample delivery systems, a fast detector *etc.* will still enable a time resolution of 1 ms for SSX measurements [Fig. 1 ▸(*c*)]. Based on the modelling performed here, the value of an EHF beamline over and above this baseline needs to be critically assessed. Undoubtedly, some samples will be able to endure the increased damage potential of 1 × 10^15^ photons s^−1^ but, as suggested above, caution should be exercised. Even if only a 10 K increase in temperature is expected, this is still 10 K more than the ‘room temperature’ that was the purpose of the experiment. The diffracted photons from the exposure will also come from an ensemble of molecules at different temperatures. These differences may ultimately prove minimal, but it is essential to perform the control experiments that will provide confidence in the results and enable the discernment of the true signal from artefacts. Fig. 8 ▸ shows some sample and beam guidelines, based on the modelling performed here, to consider when performing a high time resolution experiment.

Finally, the PAD modelling suggests that the question regarding the dose rate at synchrotrons does have a final conclusion. Ultimately, the cooling rate of a crystal, at both 100 and 293 K, will be the limiting factor that inhibits the ongoing quest for higher dose rates in protein crystallography. Essentially, to take advantage of higher dose rates for time-resolved purposes, the total dose delivered to a crystal will need to be considered if the sample is to yield undamaged diffraction. This dose can principally be reduced by using larger crystals and beams. The damage incurred at these high dose rates (GGy s^−1^) will be specific to the sample and delivery system and should be assessed at the start of an experiment so that an appropriate exposure time limit can be found and exploited successfully.

## 6. Related literature

For further literature related to the supporting information, see Deacon *et al.* (1998 ▸), Gruhl *et al.* (2023 ▸), Holton (2009 ▸), Kim & Nam (2022 ▸), Martiel *et al.* (2020 ▸), Sanchez-Weatherby *et al.* (2019 ▸), Sun *et al.* (2019 ▸) and Winter *et al.* (2019 ▸).

## Supplementary Material

Supporting information. DOI: 10.1107/S2052252525011224/car5002sup1.pdf

Simulated data: https://doi.psi.ch/detail/10.16907/e9376ecb-dc28-4cc3-9529-8a0933291e8b

## Figures and Tables

**Table 1:** Pulse and delay durations used in the modelling in this work Over the time scales of the relevant exposure times, changes to the pulse length and bunch rate do not materially alter the estimates of the model. They do, however, have an influence on the point of thermal equilibrium (see Section S1.9 in the supporting information).

Source	Pulse duration (s)	Delay duration (s)	Repetition rate (MHz)
DLS/SLS	5.0 × 10^−11^	1.95 × 10^−9^	500.0
ESRF-EBS	1.3 × 10^−11^	2.82 × 10^−9^	352.2
MAX IV	4.0 × 10^−10^	9.60 × 10^−9^	100.0
