# Supplementary material for: Modelling of radiation damage and beam-induced heating of room-temperature samples at extremely high flux MX beamlines
Source: IUCrJ. 2026 Feb 12;13(Pt 2):184–97. doi: 10.1107/S2052252525011224 (PMC12951827; doi:10.1107/S2052252525011224)
Supplement: Supplementary file 1 [file m-13-00184-sup1.pdf]

IUCrJ

Volume 13 (2026)

Supporting information for article:

Modelling of radiation damage and beam-induced heating of room-temperature samples at extremely high flux MX beamlines

Martin V. Appleby, Michal W. Kepa, Graeme Winter, Katherine E. McAuley and John H. Beale

S1. Supplementary information

S1.1. Multilayer monochromators

Since their introduction at Cornell High Energy Synchrotron Source (CHESS) in 1998 (Deacon *et al.*, 1998), double multilayer monochromators (DMMs) have not been widely applied at the world's synchrotrons. However, in the last 10 years, they have grown in popularity with their application at VMXi, Diamond Light Source (DLS) (Sanchez-Weatherby *et al.*, 2019), MPX, Shanghai Synchrotron Radiation Facility (SSRF) (Sun *et al.*, 2019), and 1C, Pohang Light Source II (PLS-II) (Kim & Nam, 2022). With the upgrade to diffraction-limited-storage ring (DLSR)s, more beamlines have taken and will take the opportunity to install a double multilayer monochromator (DMM) (Table S1).

Table S1. ***A list of third- and fourth-generation source MX beamlines that have, or plan to have, DMMs.*** *The DSLRs are indicated by a †.*

year	beamline	source
1998	FlexX	CHESS
2018	VMXi	DLS
2022	MPX	SSRF
2022	1C	PLS-II
2022	ID29	ESRF-EBS†
2024	MX3	Australian Synchrotron
2024	MicroMAX	MAX IV†
2026	PXI-VESPA	SLS 2.0†
2029	K24	Diamond II†
2029	K04	Diamond II†

The driving force behind this trend is the further increase in flux achievable by the addition of a DMM to a beamline. The multilayers increase the number of photon energies that satisfy the Bragg condition at given angles, changing the 0.01 % bandwidth of the typical Si(111) double crystal monochromator (DCM) to $0.4 - 2.0$ % and creating 'pink beam' (Figure S1). This increased acceptance increases the number of photons by perhaps another $1 - 2$ orders of magnitude depending on the bandwidth. However, it should be noted that the increased bandwidth with large unit cells can give rise to overlapping low-resolution diffraction spots that may cause integration

issues.

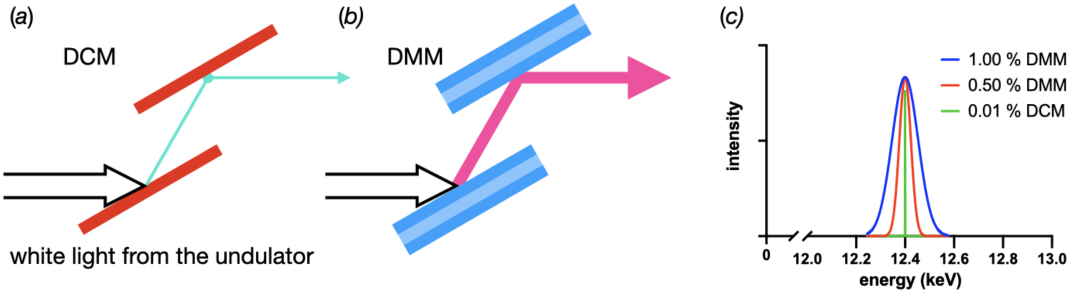

Fig. S1. **Single crystal and multi-layer monochromators.** The multi-layers of the DMM allow for multiple wavelengths (so-called pink beam) of the white light from the undulator to be utilised when compared to the DCM. This increases the usable bandwidth and hence number of photons available to be transmitted to the sample. (a) and (b) Energy selection using a DCM (red) and DMM (blue), respectively. The polychromatic, white light from the source is shown as a white arrow. (c) Modelled Gaussian curves showing the relative increase in percentage energy acceptance of a DCM at 0.01% and DMM at 0.5 and 1.0% ($\Delta E/E$).

S1.2. Calibration of `nanoBragg` parameters

S1.2.1. Production and mounting bovine insulin crystals Bovine insulin crystals were produced using a similar method as described in (Winter *et al.*, 2019). Briefly, bovine insulin (Sigma-Aldrich, I5500) was solubilised in water and its concentration was measured using a NanoDrop spectrometer (ThermoFischer). 25 mg/ml insulin solution was then mixed with 0.1 M BIS-TRIS propane pH 7.5, 0.1 M potassium iodide and 30% (w/v) PEG 3350 in a 1:1 ratio to a total volume of 400 nl in a 96-well sitting drop crystallisation plate. Hundreds of 30 µm insulin crystals were mounted on microporous membranes (Martiel *et al.*, 2020) and cryo-cooled in liquid nitrogen prior to data-collection.

S1.2.2. Data collection and processing of `nanoBragg` bench-marking data The benchmarking data were collected from the PXII beamline, Swiss Light Source (SLS), on the 04.02.2021. The approximate full flux of the beamline was 2×10^{12} photons·s^{-1}. Data were collected with a transmission of 20% that was further reduced by 70% by the addition of an aperture giving a final beam profile and flux of 30×10 µm full-width half-maximum (FWHM) and 1.16×10^{11} photons·s^{-1}, respectively. Five 60° wedge datasets using an oscillation angle of 0.1° were collected on five different crystals on the target. An example image from these data is shown in Figure S2(a). The raw images and processed data were used as guides. The number of mosaic domains and their spread were adjusted such that the generated data gave comparable spot sizes and intensities to the real data.

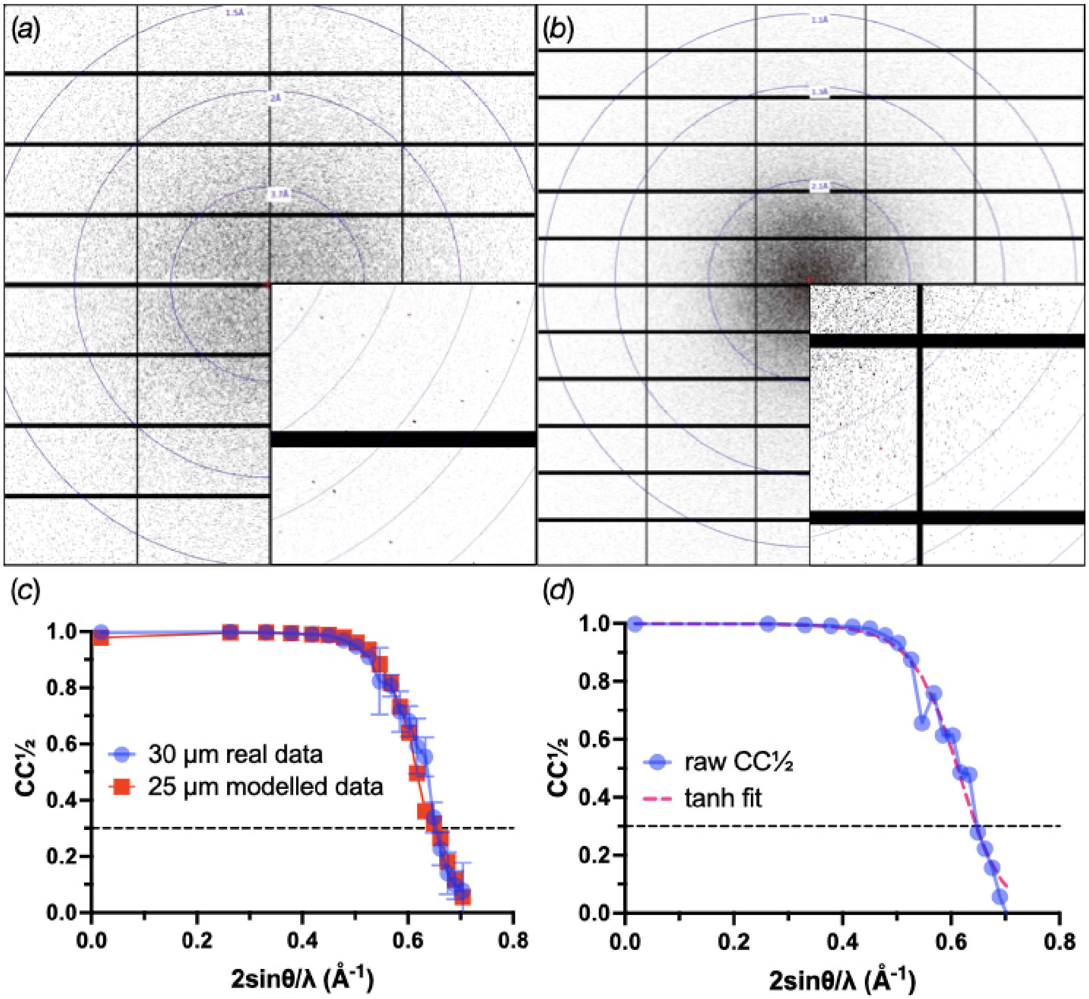

Fig. S2. **Comparison between actual diffraction data from five 30** μm **insulin crystals and generated data from a 25** μm **insulin crystal using nanoBragg.** (*a*) and (*b*) An example diffraction image from a real 30 μm and modelled 25 μm insulin crystal on an EIGER2X 16 Mpixel and PILATUS 6 Mpixel detector, respectively. 10 ms exposure times were used in both the real and simulated data. The real data were collected using a 30×10 μm beam (FWHM) with a flux of approximately 1.16×10^{11} photons·s^{-1}. (*c*) A plot showing the CC½ curves from the real and modelled data. The real data points are the mean of five 60° wedge datasets. The error bars show the 0.95 confidence interval. (*d*) An example of a *tanh* curve fit to a raw CC½ curve.

The data were processed using DIALS (version 3.12.1) suite (Beilsten-Edmands *et al.*, 2024) and followed a standard DIALS pipeline with default parameters (see

§ S1.3). An averaged CC½ curve from the five datasets is shown in Figure S2(*b*), and summary statistics are displayed in Table S2.

Table S2. ***A comparison of the data reduction statistics from five 30*** µm ***insulin crystals and generated data from a 25*** µm ***insulin crystal using*** **`nanoBragg`*****.*** *The real 30* µm *datasets were collected from five 30* µm *insulin crystal using a* 30×10 µm *beam (FWHM) with a flux of approximately* 1.16×10^{11} *photons·s*$^{-1}$ *and a 60° wedge. The real data are shown, where appropriate, as averages with a 0.95 confidence interval. The values in brackets indicate the highest resolution bin.*

	**real 30** µm	**modelled 25** µm
images	600	900
resolution	$55.14 - 1.52$ $(1.55 - 1.52)$	$77.22 - 1.52$ $(1.55 - 1.52)$
observations	120886 ± 633 (5273 ± 122)	235927 (11280)
unique	12314 ± 56 (627 ± 18)	23917 (1174)
multiplicity	9.8 ± 0.1 (8.4 ± 0.1)	9.9 (9.6)
completeness (%)	100 (100)	100 (100)
Mean I/σ	11.680 ± 1.627 (0.480 ± 0.104)	2.7 (0.2)
R_{merge}	0.083 ± 0.011 (1.816 ± 0.268)	0.170 (3.337)
R_{meas}	0.088 ± 0.011 (1.935 ± 0.286)	0.180 (3.526)
R_{pim}	0.028 ± 0.004 (0.662 ± 0.096)	0.060 (1.132)
CC½	0.9988 ± 0.001 (0.409 ± 0.069)	0.982 (0.368)

S1.3. DIALS processing bash scripts

Stills processing using **xia2.ssx**

```
#!/bin/bash

module load DIALS/3.12.1

xia2.ssx \\
 template=images_*.cbf \\
 dials_import.phil=import.phil \\
 space_group=I213 \\
 unit_cell=77,77,77,90,90,90 \\
 reference=insulin.pdb \\
 d_min=1.1 \\
 spotfinding.phil=findspot.phil
```

Example import.phil

```
filter.min_spot_size=1
threshold.dispersion.global_threshold=???? # required for higher flux and
    exposure time varients
spotfinder {
  threshold {
    algorithm = *dispersion dispersion_extended radial_profile
    dispersion {
      gain = 1
      min_local = 2
      global_threshold = 25000
    }
  }
}
```

Rotation processing using standard **dials**

```
#!/bin/bash

module load DIALS/3.12.1

dials.import *.cbf
dials.find_spots \\
  imported.expt \\
  threshold.dispersion.global_threshold=????? # required for higher flux
      and exposure time varients
dials.index \\
  imported.expt \\
  strong.refl \\
  space_group=P213 \\
  unit_cell=77,77,77,90,90,90
dials.refine indexed.*
dials.integrate refined.*
dials.scale integrated.*
```

S1.4. RADDOSE-3D input files

The comments coloured in green and preceded by a '#' highlight the different RADDOSE-3D inputs that were changed to model the different crystal sizes, fluxes, and beam parameters.

Insulin input file used in nanoBragg modelling and initial PAD models

```
##########################################################################
#                            Crystal Block                               #
##########################################################################

Crystal
Type Spherical
Dimensions ? ? ?    # ? = a,b,c length, e.g., 1 1 1 for 1 um cube
PixelsPerMicron ?   # 20/a,b,c length, e.g., 1 um xtal = 20/1

AbsCoefCalc  Exp
SUBPROGRAM MONTECARLO
Runs 10
SIMPHOTONS 10000000

PDB 3I40.pdb
SolventHeavyConc Na 0.1

GONIOMETERAXIS 0
CALCSURROUNDING TRUE
SURROUNDINGHEAVYCONC Na 100
SURROUNDINGTHICKNESS ? ? ?    # ? = a,b,c length, e.g., 1 1 1 for 1 um cube

##########################################################################
#                               Beam Block                               #
##########################################################################

Beam
Type Tophat
Flux ?                        # 1e11, 1e12, 1e13, 1e14 or 1e15
ENERGY 12.4
Collimation Rectangular ?     # ? = a,b,c length, e.g., 1 1 for 1 um cube
ENERGYFWHM 0.0496             # 0.4 % bandwidth for 1e14 or 1e15

##########################################################################
#                               Wedge Block                              #
##########################################################################

Wedge 0 ?             # ? = either 0 or 900, depending on still or rotation
ExposureTime ?        # ? = depends on still/rotation and desired time
AngularResolution 2   # commented out for still images
```

Ruby crystal input file

```
###########################################################################
#                            Crystal Block                                #
###########################################################################

Crystal
Type Spherical
Dimensions 40 40 40
PixelsPerMicron 2

AbsCoefCalc SMALLMOLE
SMALLMOLEATOMS Al 0.33 Cr 0.00333 O 0.5
UNITCELL 4.75 4.75 12.99
NUMMONOMERS 12

###########################################################################
#                               Beam Block                                #
###########################################################################

Beam
Type Gaussian
Flux 3.18e12
FWHM 20 20
ENERGY 9.2
Collimation Rectangular 100 100

###########################################################################
#                               Wedge Block                               #
###########################################################################

Wedge 0 0
ExposureTime 1
```

Rhodopsin input file used in the ID29 example

```
##########################################################################
#                           Crystal Block                                #
##########################################################################

Crystal
Type Cube
Dimensions 5 5 5
PixelsPerMicron 4

AbsCoefCalc  Exp
SUBPROGRAM MONTECARLO
Runs 10
SIMPHOTONS 10000000

PDB 7zbc.pdb

GONIOMETERAXIS 0
CALCSURROUNDING TRUE
SURROUNDINGTHICKNESS ? ? ?    # ? = a,b,c length, e.g., 1 1 1 for 1 um cube

##########################################################################
#                              Beam Block                                #
##########################################################################

Beam
Type Gaussian
Flux 1e15
FWHM 4 2
ENERGY 12.4
Collimation Rectangular 200 200 # assumed no slitting of beam at focus
ENERGYFWHM 0.0578

##########################################################################
#                              Wedge Block                               #
##########################################################################

Wedge 0 0
ExposureTime 1        # to calculate total dose in 1 s for PAD model
```

Haemoglobin input file used in the MAXIV example

```
##########################################################################
#                          Crystal Block                                 #
##########################################################################

Crystal
Type Cube
Dimensions ? ? ?                # ? = a,b,c length
PixelsPerMicron ?               # 20/a,b,c length, e.g., 1 um xtal = 20/1

AbsCoefCalc  Exp
SUBPROGRAM MONTECARLO
Runs 10
SIMPHOTONS 10000000

PDB 7dy3.pdb
SolventHeavyConc Li 0.2

GONIOMETERAXIS 0
CALCSURROUNDING TRUE
SURROUNDINGHEAVYCONC Li 200
SURROUNDINGTHICKNESS 10 10 10

##########################################################################
#                               Beam Block                               #
##########################################################################

Beam
Type Gaussian
Flux ?                          # 1e12, 1e13, 1e14, or 1e15
FWHM ? ?                        # either xtal width or 0.589*xtal wdith
                                # to mimic matching of xtal to FW of beam
ENERGY 12.4
Collimation Rectangular 200 200 # assumed no slitting of beam at focus
ENERGYFWHM 0.0578

##########################################################################
#                               Wedge Block                              #
##########################################################################

Wedge 0 0
ExposureTime 1        # to calculate total dose in 1 s for PAD model
```

S1.5. Modelling crystal heating and cooling

This section contains tabulations of the various constants used in the generation of the pulsed-adiabatic decay (PAD) model.

S1.5.1. Heat capacity of lysozyme A key parameter in the PAD model is the heat capacity (C_p) of a protein crystal as a function of temperature and water content. The only published paper that experimentally measures these is from Miyazaki *et al.* (2000). Table S3 lists the measured C_p values for lysozyme with different moisture contents at 100 and 293 K. The default PAD model assumes a water content of 45.7%.

Table S3. ***Miyazaki* et al. *(2000) experimentally measured the change in lyzozyme crystal C_p as a function of temperature and water content.*** *For clarity, this table displays the formula mass of the lysozyme with different percentages of water contents (M) and the C_p in kJ·K^{-1}·mol^{-1} as reported by Miyazaki* et al. *(2000). It also displays the calculated C_p in J·kg^{-1}·K^{-1} from these two values.*

		C_p (kJ·K^{-1}·mol^{-1})		C_p (J·kg^{-1}·K^{-1})	
**H_2O content (%)**	**M (kg·mol^{-1})**	**100 K**	**300 K**	**100 K**	**300 K**
45.7	27.3	18.9	73.6	692.5	2,693.7
41.0	25.1	16.8	64.5	669.3	2,564.7
36.4	23.3	15.4	57.0	659.6	2,443.0
31.6	21.7	13.9	50.3	640.8	2,316.8
24.0	19.5	11.9	41.3	609.2	2,116.7
13.6	17.2	9.7	30.2	563.8	1,760.0
7.4	16.0	8.6	23.8	539.1	1,484.4
-	14.8	7.4	18.4	500.2	1,242.1

S1.5.2. Other constants The physical constants used to calculate the point of thermal equilibrium *via* the KKT model and to calculate the thermal decay in the PAD model.

Table S4. ***Tabulation of other constants required for the KKT and PAD models.***

v (m^2 s^-1)	kinematic viscosity of gas	1.90×10^{-6}<br>1.50×10^{-5}	100 K N_2 cryo-stream<br>300 K air
κ (W m^{-1} K^{-1})	thermal conductivity of gas	9.80×10^{-3}<br>2.60×10^{-2}	100 K N_2 cryo-stream<br>300 K air
μ (m s^{-1})	gas velocity	3.30<br>1.01	100 K N_2 cryo-stream<br>300 K air
ρ (kg m^{-3})	density	1.47×10^{3}<br>3.98×10^{3}<br>2.50×10^{3}	protein<br>ruby<br>glass
C_p (J K^{-1} kg^{-1})	heat capacity	6.92×10^{2}<br>2.69×10^{3}<br>7.50×10^{2}<br>8.40×10^{2}	100 K 45.7% H_2O protein<br>300 K 45.7% H_2O protein<br>300 K ruby<br>300 K glass

S1.6. Example decay and heating parameters for spherical insulin crystals

Table S5. ***Dose rates calculated in RADDOSE-3D for spherical insulin crystals at different fluxes.***

**crystal**	**Dose rate by flux (GGy·s^{-1})**			
**diameter** (µm)	1×10^{12}	1×10^{13}	1×10^{14}	1×10^{15}
100	6.03×10^{-5}	6.05×10^{-4}	6.03×10^{-3}	6.05×10^{-2}
50	2.41×10^{-4}	2.41×10^{-3}	2.41×10^{-2}	2.40×10^{-1}
25	9.42×10^{-4}	9.39×10^{-3}	9.52×10^{-2}	9.40×10^{-1}
10	5.29×10^{-3}	5.28×10^{-2}	5.21×10^{-1}	5.33
5	1.40×10^{-2}	1.42×10^{-1}	1.43	14.20
1	9.42×10^{-2}	9.49×10^{-1}	9.40	93.60

Table S6. ***Modelled cooling constants for spherical insulin crystals with a solvent content of 45.7% in 293 K air.*** *The constants below assume a 500 MHz pulse rate and 50 ps pulse duration.*

**crystal**			**ΔT by flux (K)**		
**diameter** (µm)	**k**	**half life (s)**	1×10^{12}	1×10^{13}	1×10^{15}
1	8,690.0	7.97×10^{-5}	5.30×10^{-5}	5.46×10^{-4}	5.35×10^{-2}
5	7,77.0	8.92×10^{-4}	9.28×10^{-6}	9.23×10^{-5}	9.26×10^{-3}
10	275.0	2.52×10^{-3}	3.00×10^{-6}	3.00×10^{-5}	3.00×10^{-3}
25	69.5	9.97×10^{-3}	5.27×10^{-7}	5.27×10^{-6}	5.26×10^{-4}
50	24.6	2.82×10^{-2}	1.34×10^{-7}	1.34×10^{-6}	1.34×10^{-4}
100	8.7	7.97×10^{-2}	3.35×10^{-8}	3.35×10^{-7}	3.35×10^{-5}

S1.7. Assessing the fit of the PAD against experimental data

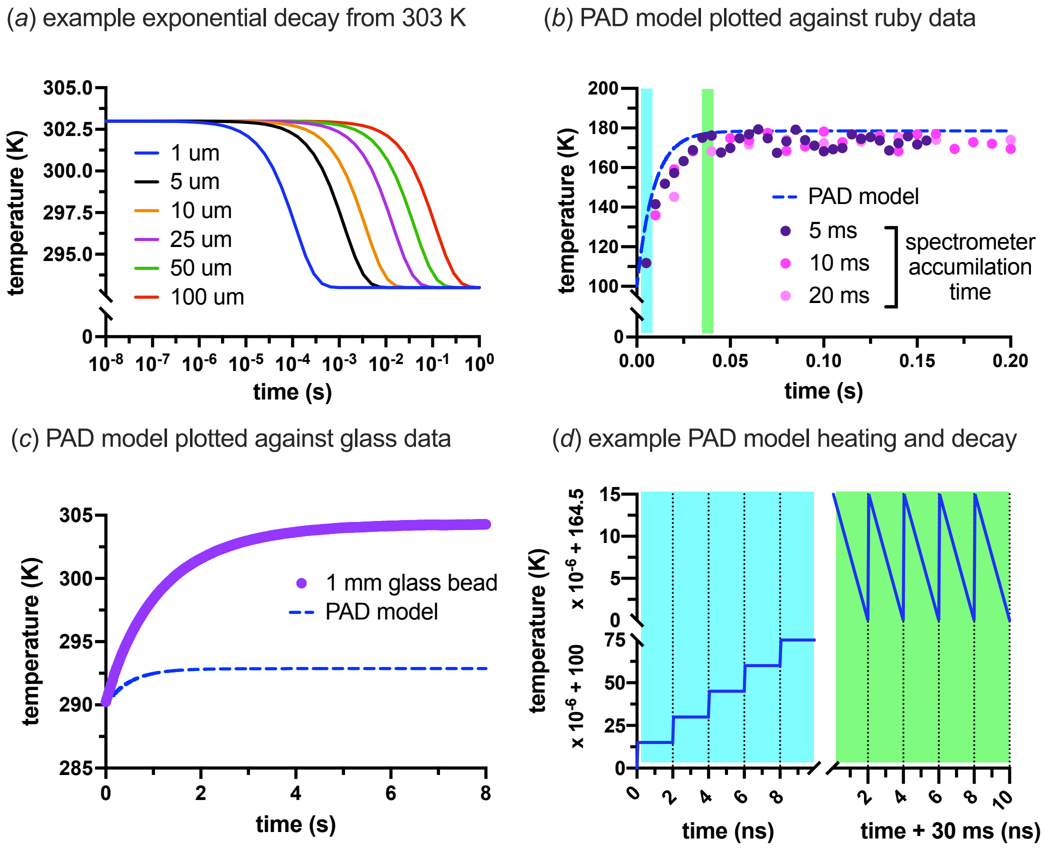

Fig. S3. **Construction of PAD model for time dependent heating of crystals *via* an X-ray beam in a gaseous environment.** (*a*) Example exponential decay curves for different sized protein crystals from 303 K back to 293 K. (*b*) and (*c*) Comparison of the PAD model to the raw data collected from the ruby crystals and glass beads measured by Warren *et al.* (2019) and Snell *et al.* (2007), respectively. Raw data from both has been shown with the authors' permission. (*b*) Time dependent changes in a 40 µm spherical ruby crystal exposed to a 20 × 20 µm (FWHM), 9.2 keV X-ray beam with a flux of $3.18{\times}10^{12}$ photons·s^{-1} at three different accumulation times (5, 10 and 20 ms). (*c*) Time dependent changes between a 1 mm glass bead exposed to a 103 × 84 µm (FWHM), 6.5 keV X-ray beam with a flux of $3.24{\times}10^{12}$ photons·s^{-1} under a continuous exposure. (*d*) A zoomed in view of the ruby PAD model at the first 0 – 10 ns [cyan rectangle in (*b*)], and at the point of thermal equilibrium (30 ms). The points of the 50 ps X-ray pulses are marked by vertical lines. The heat is allowed to decay exponentially over the following 1.95 ns. As can be observed, the PAD model reaches an equilibrium when the decay eventually equals the heat from the absorbed energy [green rectangle in (*b*)].

S1.8. Full heating curves for the 293 K data assuming a tophat beam profile, 500 MHz source and a 0.4% bandwidth.

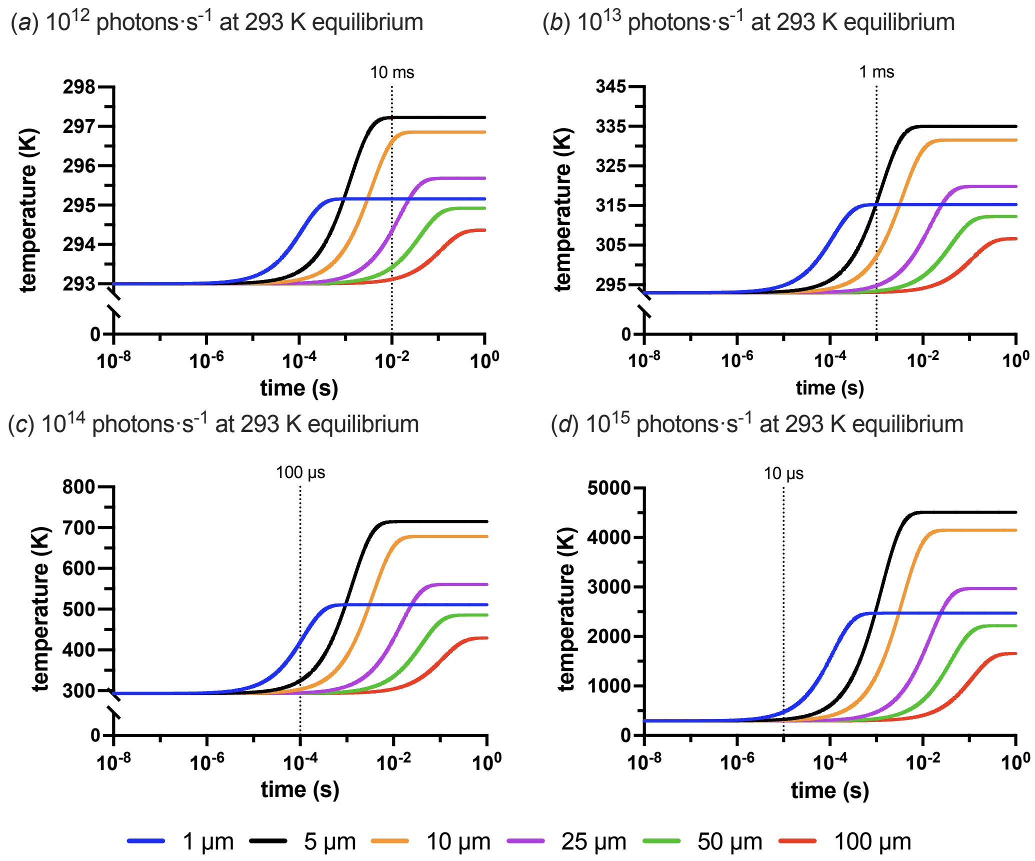

Fig. S4. **PAD modelling of the thermodynamic equilibrium for different crystal sizes and beam fluxes.** (*a*), (*b*), (*c*) and (*d*) Spherical crystal freely suspended in a N_2 gas stream at 293 K with 1×10^{12}, 1×10^{13}, 1×10^{14} and 1×10^{15} photon flux, respectively. The nominal exposure time to deliver 1×10^{10} photons for each of the different fluxes is also shown.

S1.9. Comparison of the influence on source repetition rate on PAD model results

In this example, the three source pulse durations and repetition rates from ID29, MicroMAX and PXI (Table 1 in the main paper) were used to calculate the point of thermal equilibrium and ΔT in 10 µs, assuming a total dose of 150 kGy over the 10 µm. The crystal diameter was 10 µs and the total dose in 1 s was 1.5×10^4 MGy. Figure S5(*a*) shows that there is a small change in the calculated point of thermal equilibrium between the different sources. However, Figure S5(*b*) shows that, on the time scale of the desired 150 kGy exposure, there is no meaningful difference in the estimated ΔT.

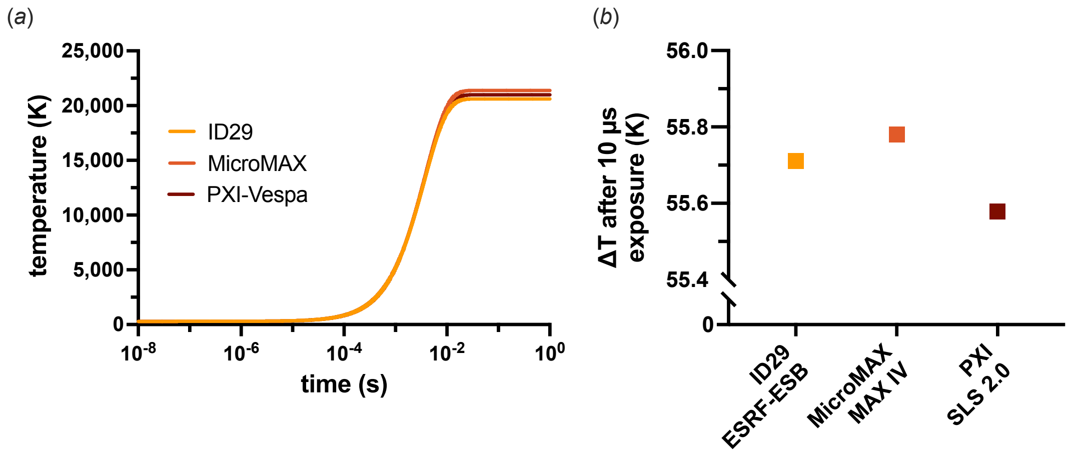

Fig. S5. **Modelling the influence of source repetition rate on PAD model results.** (*a*) PAD modelling of the thermodynamic equilibrium, using the different source parameters of the ESRF-EBS, MAXIV and the SLS 2.0, of a 10 µm crystal with a total dose over 1 s of 1.5×10^4 MGy. (*b*) The estimated ΔT rise after 10 µs and a dose of 150 kGy at the sources modelled in (*a*).

S1.10. Calculated dose rates for 5 µm *rhodopsin crystal using* `RADDOSE-3D`

Table S7. ***Calculated doses using* `RADDOSE-3D` *for 5*** µm ***bovine rhodopsin crystals at ID29.*** *The* `RADDOSE-3D` *input file is given in Section S1.4 and used Protein Data Bank (PDB):7ZBC (Gruhl* et al.*, 2023) as the model. The modelling assumed a Gaussian beam profile of* 4×2 µm *with essentially no collimation and a 1% energy bandwidth.*

**flux (photons·s^{-1})**	**dose rate (GGy·s^{-1})**
1×10^{12}	0.010
1×10^{13}	0.104
1×10^{14}	1.039
1×10^{15}	10.360

S1.11. Calculated dose rates for haemoglobin crystals using `RADDOSE-3D`

Table S8. ***Calculated doses using* `RADDOSE-3D` *for haemoglobin crystals at MicroMAX.*** *The* `RADDOSE-3D` *input file is given in Section S1.4 and used PDB:7DY3 as the model. The modelling assumed a Gaussian beam profile with essentially no collimation and a 1% energy bandwidth. In the first run, the crystal size was matched to the FWHM. In the second run, it was matched to the FW, here assumed to be* $1.699 \times FWHM$.

	**first run**		**second run**	
**FWHM** (µm)	**crystal size** (µm)	**dose rate (GGy·s^{-1})**	**crystal size** (µm)	**dose rate (GGy·s^{-1})**
25	25	0.359	42.5	0.196
20	20	0.556	34.0	0.305
15	15	0.986	25.5	0.541
10	10	2.136	17.0	1.200
5	5	7.442	8.5	4.469

S1.12. Assessment of necessary crystal translation speeds to limit beam-induced heating

Table S9. ***The times taken, based on the insulin crystal PAD modelling, for a crystal illuminated by a given beam size to increase to a given temperature at a flux of*** 1×10^{15} ***photons·s***$^{-1}$***.***

**beam/crystal diameter** (µm)	**time to reach ΔT** (µs) **10 K**	**5 K**	**1 K**
1	2.04	0.49	0.08
5	4.93	2.31	0.44
10	14.10	6.74	1.30
25	76.49	36.78	7.14
50	297.06	142.83	27.74
100	1,176.27	565.56	110.15

Table S10. ***The crystal length required, based on the times given in Table S9, such that a single crystal can be continuously illuminated with*** 1×10^{15} ***photons·s***$^{-1}$ ***for 10*** µs ***and the temperature rise limited to either 10, 5 or 1 K.***

**beam/crystal diameter** (µm)	**minimum crystal length** (µm) **10 K**	**5 K**	**1 K**
1	4.91	20.26	129.75
5	10.14	21.61	113.14
10	7.09	14.84	76.80
25	3.27	6.80	35.00
50	1.68	3.50	18.02
100	0.85	1.77	9.08

S1.13. Practically using the PAD model.

The PAD model and `nanoBragg` scripts are publicly available from Gitea[1].

Required python modules

The model is written in `python` and is run from the command line and requires the following packages:

- `pandas`
- `numpy`
- `matplotlib`
- `os`
- `errno`
- `regex`
- `argparse`
- `scipy`
- `loguru`

Minimum variables

The variables that must always be defined when running for model are:

- '-n' name of run. This will be propagated as the name used in a folder containing the output files and as a prefix. Default is 'pad-model'.
- '-e' the temperature of the surrounding environment. Only 100 or 293 K are accepted.
- the shortest dimension of the crystal/s in question. This can be defined directly in the command line or in a parameter file.
- '-s' the crystal shape. Only 'cube' or 'sphere' are accepted. Default is 'sphere'.
- '-p' X-ray pulse duration in seconds. Default is 5×10^{-11} (50 ps).

[1] https://gitea.psi.ch/crystal_heating

- '-d' delay between adjacent pulses. Default is 1.95×10^{-9} (1.95 ns).

Additional inputs may be required depending on whether the crystal sizes are defined from the command line or if a parameter file is supplied, and these cases are described below.

Model modes

The model essentially has two modes: a 'quick' and 'full' pertaining to the speed and accuracy the user requires. The default mode is 'full' PAD that necessitates the full modelling of every heating and cooling cycle up to a user-defined end point ('-t' in seconds). This can take a long time if many different crystals are run concurrently and up to the second time scale. The alternative is to run in 'quick' mode ('-q' True). Here, it is assumed that the KKT equation [equation (6) in Section 3.2 of the main paper] can reasonably estimate the point of thermal equilibrium (T_{max}). Knowing this, only the exponential rate constant (k) [see below, equation (S3)] is required to be able to fit an exponential plateau curve [equation (S1)].

$$T = T_{max} - (T_{max} - T_{min}) \cdot \exp(-kt) \tag{S1}$$

The result is very fast and can be a useful starting point. It also usually produces very similar heating estimates to the full model for shorter exposure times.

Defining crystal sizes from the command line

The first step when using the model is to define the crystal dimensions that are being investigated and, hence, how fast the crystal will cool during each cooling cycle and defining the dose that will be deposited in the crystal during each heating cycle. Practically, this can either be done from the command line by asking the model to estimate the dose, or a parameter file can be provided where an external program, such as `RADDOSE-3D` has been used.

If the crystal sizes are defined from the command line, with an input such as the following:

```
$ python pad_model.py -l 25,50,100 -f 1e15 -e 293 -q True
```

crystal lengths of 25, 50 and 100 will be modelled. Here the '-l' flag expects a list of shortest crystal dimensions separated by a comma. The flux ('-f') is now also required as the model will now estimate the accumulated dose in the crystal using the formulae given in Holton (2009) [equation (S2)].

$$\text{Dose rate } (\text{Gy}\cdot\text{s}^{-1}) \approx \frac{\text{X-ray fluence}}{2000} \tag{S2}$$

The model will assume that the X-ray beam profile is the same size as the crystal length given by '-l' flag. This approximation seems to hold reasonably true for crystal sizes > 25 µm, with no significant heavy atom content and an incident energy of 12.4 keV. Significant deviations will occur from programs such RADDOSE-3D when crystal lengths get smaller, as non-linear processes such as the escape of photo-electrons outside of the crystal volume need to be taken into account.

The heating constants are then estimated from the dose rate ($\text{MGy}\cdot\text{s}^{-1}$), the pulse duty cycle and the Cp [equation (5) in Section 2.4.2 of the main paper]. The cooling h parameter has been hardcoded for samples at either 100 or 293 K *via* equation (4) in Section 2.4.1 of the main paper. From this, and the crystal surface area (A), mass (m) and Cp, the decay rate constant (k) can be estimated using equation (S3).

$$k = \frac{h \cdot A}{Cp \cdot m} \tag{S3}$$

Defining crystal sizes and deposited dose using an input parameter file

The parameter file needs to be in a comma separated format. An example of an input parameter file is:

```
length,100,50,25,10,5,1
dose_1s,43.43,173.04,675.44,3817.55,11846.03,78350.60
shape,cube,cube,cube,cube,cube,cube
```

Here, the crystal lengths that will be modelled are given in the first row. Dose rates in MGy·s^{-1} and crystal shapes can be added in subsequent rows, although this is not essential. If the desired shapes are all the same, such as in the example above, this can be specified in the command line.

Outputs

The model will produce a series of outputs in a folder labelled after the input name argument, and this will also be the prefix of all the output files. These are:

- run_name.log - log file of run
- run_name_raw.csv - full output of pad model - only output if '-w' True
- run_name_reduced.csv - log sample of raw output
- run_name_fit_parameters.csv - parameters from exponential plateau fit to reduced data
- run_name.png - plot of pad prediction against reduced data
- run_name_fit_curve.csv - log sample of fit curve
- run_name_exposure.csv - predicted crystal temperatures at nominal exposure times.

Example 1

The first example runs the PAD model in ‘quick’ mode at 100 K and uses the Holton dose approximation for crystals with shortest dimensions of 25, 50 and 100 µm at 1×10^{13} photons·s^{-1}. The raw output results are shown in Figure S6.

```
$ python pad_model.py -l 25,50,100 -f 1e13 -e 100 -q True
```

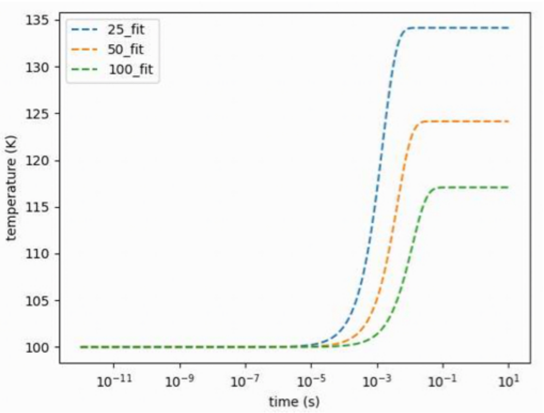

time (s)	25	50	100
1.0E-06	100.0	100.0	100.0
1.0E-05	100.2	100.1	100.0
1.0E-04	102.2	100.6	100.1
1.0E-03	116.7	105.1	101.4
1.0E-02	134.1	121.9	109.7
1.0E-01	134.1	124.1	117.1
1.0E+00	134.1	124.1	117.1
1.0E+01	134.1	124.1	117.1

Fig. S6. **Results from the first example.** A heating after an exposure of 1×10^{10} photons has been highlighted.

Example 2

This is the command line sequence that was used to generate the data presented in Figure 6 of the main paper. The content of the parameter file is shown below. To try and increase the accuracy of the model for MicroMAX, the X-ray pulse and delay duration have been added as arguments. The raw output results are shown in Figure S7.

```
$ python pad_model.py -n maxiv-fwhm -e 293 -p fwhm-constants.csv -t 0.1 -s
    cube -x 400E-12 -d 9.6E-9
```

```
length,25,20,15,10,5
dose_1s,359.1601225,556.82,986.01,2135.70,7441.61
```

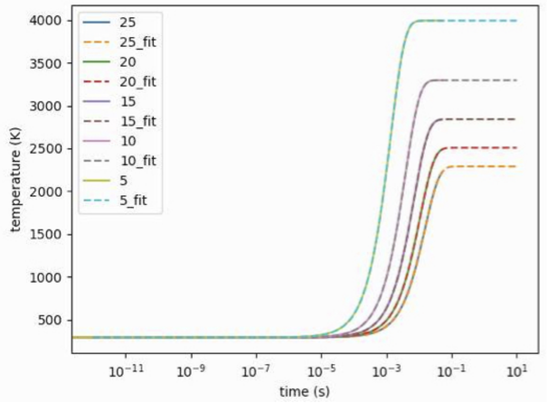

time (s)	25	20	15	10	5
1.0E-06	293.1	293.2	293.4	293.8	295.8
1.0E-05	294.3	295.1	296.7	300.9	320.5
1.0E-04	306.3	313.6	329.3	371.2	559.2
1.0E-03	422.0	490.4	634.0	989.8	2239.3
1.0E-02	1265.6	1636.8	2235.0	3081.8	3990.4
1.0E-01	2286.8	2507.4	2840.3	3296.1	3992.5
1.0E+00	2289.3	2507.6	2840.3	3296.1	3992.5
1.0E+01	2289.3	2507.6	2840.3	3296.1	3992.5

Fig. S7. **Results from the second example.** A heating after an exposure of 1×10^{10} photons has been highlighted.

References

Beilsten-Edmands, J., Parkhurst, J. M., Winter, G. & Evans, G. (2024). *Methods in Enzymology*, **709**.
https://pubmed.ncbi.nlm.nih.gov/39608945/

Deacon, A. M., Appleby, T., Bilderback, D. H., Ealick, S. E., Fontes, E. & Thiel, D. J. (1998). *Journal of Synchrotron Radiation*, **5**(3), 494–496.
//scripts.iucr.org/cgi-bin/paper?he3134

Gruhl, T., Weinert, T., Rodrigues, M. J., Milne, C. J., Ortolani, G., Nass, K., Nango, E., Sen, S., Johnson, P. J., Cirelli, C., Furrer, A., Mous, S., Skopintsev, P., James, D., Dworkowski, F., Båth, P., Kekilli, D., Ozerov, D., Tanaka, R., Glover, H., Bacellar, C., Brünle, S., Casadei, C. M., Diethelm, A. D., Gashi, D., Gotthard, G., Guixà-González, R., Joti, Y., Kabanova, V., Knopp, G., Lesca, E., Ma, P., Martiel, I., Mühle, J., Owada, S., Pamula, F., Sarabi, D., Tejero, O., Tsai, C. J., Varma, N., Wach, A., Boutet, S., Tono, K., Nogly, P., Deupi, X., Iwata, S., Neutze, R., Standfuss, J., Schertler, G. & Panneels, V. (2023). *Nature*, **615**(7954), 939–944.
https://www.nature.com/articles/s41586-023-05863-6

Holton, J. M. (2009). *Journal of Synchrotron Radiation*, **16**(2), 133–142.

Kim, Y. & Nam, K. H. (2022). *Crystals*, **12**(11), 1637.
https://www.mdpi.com/2073-4352/12/11/1637/htm https://www.mdpi.com/2073-4352/12/11/1637

Martiel, I., Buntschu, D., Meier, N., Gobbo, A., Panepucci, E., Schneider, R., Heimgartner, P., Müller, D., Bühlmann, K., Birri, M., Kaminski, J. W., Leuenberger, J., Oliéric, V., Glettig, W. & Wang, M. (2020). *Journal of Synchrotron Radiation*, **27**(3), 860–863.

Miyazaki, Y., Matsuo, T. & Suga, H. (2000). *Journal of Physical Chemistry B*, **104**(33), 8044–8052.
https://pubs.acs.org/doi/abs/10.1021/jp0007686

Sanchez-Weatherby, J., Sandy, J., Mikolajek, H., Lobley, C. M., Mazzorana, M., Kelly, J., Preece, G., Littlewood, R. & Sørensen, T. L. (2019). *Journal of Synchrotron Radiation*, **26**(1), 291–301.
https://scripts.iucr.org/cgi-bin/paper?xe5041

Snell, E. H., Bellamy, H. D., Rosenbaum, G. & Van Der Woerd, M. J. (2007). *Journal of Synchrotron Radiation*, **14**(1), 109–115.

Sun, B., Wang, Y., Liu, K., Wang, Q. & He, J. (2019). In *AIP Conference Proceedings*, vol. 2054.
https://doi.org/10.1063/1.5084659

Warren, A. J., Axford, D. & Owen, R. L. (2019). *Journal of Synchrotron Radiation*, **26**(4), 991–997.
https://journals.iucr.org/paper?gm5057

Winter, G., Gildea, R. J., Paterson, N. G., Beale, J., Gerstel, M., Axford, D., Vollmar, M., McAuley, K. E., Owen, R. L., Flaig, R., Ashton, A. W. & Hall, D. R. (2019). *Acta crystallographica. Section D, Structural biology*, **75**(Pt 3), 242–261.
http://www.ncbi.nlm.nih.gov/pubmed/30950396
